# Metabolomics Analysis in Different Development Stages on SP0 Generation of Rice Seeds After Spaceflight

**DOI:** 10.3389/fpls.2021.700267

**Published:** 2021-06-30

**Authors:** Deyong Zeng, Jie Cui, YiShu Yin, Yi Xiong, Mengyao Liu, Shuanghong Guan, Dayou Cheng, Yeqing Sun, Weihong Lu

**Affiliations:** ^1^Department of Food Science and Engineering, School of Chemistry and Chemical Engineering, Harbin Institute of Technology, Harbin, China; ^2^National and Local Joint Engineering Laboratory for Synthesis, Transformation and Separation of Extreme Environmental Nutrients, Harbin Institute of Technology, Harbin, China; ^3^Dalian Maritime University, Environmental Systems Biology Institute, Dalian, China

**Keywords:** rice, metabolomics, spaceflight, SJ-10 returning satellite, tricarboxylic acid cycle, amino acid metabolism

## Abstract

Spaceflight is a special abiotic stress condition. In recent years, it has been confirmed that the spaceflight caused the stress response of rice seeds, and the protein level, transcription level, and methylation level will change during the planting process after returning to the ground. However, the changes at the metabolome level are not very clear. In this study, two kinds of rice seeds, Dongnong423 (DN3) and Dongnong416 (DN6), were carried on the ShiJian-10 retractable satellite (SJ-10) for 12.5 days in orbit, returned to the ground and planted in the field until the three-leaf (TLP) and tillering stage (TS). The results of antioxidant enzyme activity, soluble sugar, and electron leakage rate revealed that the spaceflight caused the stress response of rice. The TLP and TS of DN3 identified 110 and 57 different metabolites, respectively, while the TLP and TS of DN6 identified 104 and 74 different metabolites, respectively. These metabolites included amino acids, sugars, fatty acids, organic acids and secondary metabolites. We used qRT-PCR technology to explore the changes of enzyme genes in the tricarboxylic acid cycle (TCA) and amino acid metabolism pathway. Combined with the results of metabolomics, we determined that during the TLP, the TCA cycle rate of DN3 was inhibited and amino acid metabolism was activated, while the TCA cycle rate of DN6 was activated and amino acid metabolism was inhibited. In TS, the TCA cycle rate of DN3 was inhibited, and amino acid metabolism was not significantly changed, while the TCA cycle rate of DN6 was activated and amino acid metabolism was inhibited. These results suggested that the response mechanisms of the two different rice strains to spaceflight stress are different, and these differences may be reflected in energy consumption and compound biosynthesis of rice in different growth and development stages. This study provided new insights for further exploring the effects of spaceflight.

## Introduction

The development of space science and technology as well as the development and utilization of the space field have promoted the development of space life science. In the past few decades, rice, *Arabidopsis*, corn, fruit flies, and mice have become the main model organisms for studying space life science. The space environment mainly included factors such as microgravity, radiation, circadian rhythm disturbance, weak magnetic field (Buchheim et al., 2019). Therefore, the creatures in the flight capsule would be subjected to a special abiotic stress. Previous studies have proved that plants can adapt to the spaceflight by reshaping their own metabolic network (Choi et al., 2019; Barker et al., 2020), which provided the possibility for human to use plants as a reliable food source in future long-term space missions.

Low Earth orbit (LEO) radiation was mainly composed of high energy distant galactic cosmic rays (GCR), solar particles (SEP), radiation belts, South Atlantic anomaly belts (SAA), albedo neutrons, and protons from the Earth’s atmosphere (Chancellor et al., 2014). The SJ-10 satellite had an orbital altitude of 252 Km, and the radiation absorbed dose rate and dose equivalent rate were, respectively, 0.072 mGy/d and 0.162 mSv/d (Zhou et al., 2018). The orbital altitude of the International Space Station was 370–403 km, and the radiation absorbed dose rate and dose equivalent rate were 0.2–0.3 mGy/d and 0.4–0.6 mSv/d (Zhou et al., 2018). Therefore, the International Space Station, SJ-10 and STS space missions were all in low-Earth orbit and exposed to the same types of particles. Our previous research found that short-term close-to-earth flight would cause changes in plant redox homeostasis, stress response proteins, energy metabolism, amino acid metabolism, hormone metabolism, transcription modification, and other related protein expression changes (Ma et al., 2007; Wang et al., 2008; Deyong et al., 2020; Zeng et al., 2020), and similar results have been found in ISS (Sugimoto et al., 2014; Ferl et al., 2015; Kruse et al., 2020). In the process of spaceflight, plants would be subjected to extremely severe abiotic stress (Yurkevich et al., 2018), which would lead to reprogramming of the plant’s genome, thereby affecting plant growth, development and yield (Musgrave and Kuang, 2003; De Micco et al., 2014). Previous studies have indicated that the cell wall composition of *Arabidopsis thaliana* changed in the spaceflight (Hoson et al., 2014). At the same time, the results of transcriptomic revealed that the oxidative stress signal pathway of *Arabidopsis thaliana* was significantly changed in the spaceflight, and the oxidative stress signal was used to induce the expression of molecular chaperones to protect cells from oxidative damage (Choi et al., 2019). After the *Eruca sativa* seeds that stayed on the International Space Station for 6 months and returned to the ground for planting, the emergence and growth of the seedlings were slightly delayed, and the senescence sensitivity was increased. Moreover, the transcript abundance of genes related to transcription and translation processes were reduced (Chandler et al., 2020). Other studies have proved that changes in barley leaf color and photosynthesis rate after spaceflight were related to chloroplast mutations (Jiayu et al., 2018). These results showed that the spaceflight can affect the growth and development of plants. But unfortunately, the orbital height and inclination of the aircraft were different during each spaceflight, which may cause certain differences in results. Although the conditions and methods were different each time, it has been found that there were some overlapping biochemical pathways in spaceflight, which were regulated differently. Those pathways involved in heat shock, stress response, cell wall modification and cell defense (Johnson et al., 2017). In addition, the current research on how plants respond to the spaceflight was mostly based on transcriptomics and proteomics, while the results based on metabolomics have rarely been reported.

Rice (*Oryza sativa* L.) is one of the most necessary cereal crops all over the world, and nearly half of the world’s population used it as a staple food (Shen et al., 2018). It was also a model plant for molecular biology research (Chen et al., 2019). Since 1996, our team has carried more than 50 rice varieties in different orbits of recoverable satellites or spacecraft, such as “JB-1,” “Chinese 20th recoverable satellite mission,” “Chinese 21th recoverable satellite mission,” “Shenzhou 3,” “Shenzhou 4,” and “Shenzhou 6.” After returning to the ground and planted, we analyzed phenotypic variation, cytological effects, genome mutation characteristics, and protein level changes at different growth stages (Wei et al., 2006; Sun et al., 2019). Analysis of the results showed that different orbital spaceflight can cause biological effects of rice seeds. In the STS-95 mission (The mission’s objectives involved investigating life-sciences experiments), it was found that under the stimulation of the spaceflight, the cellulose in the roots, matrix polysaccharides and root growth of rice changed (Hoson et al., 2003). Ou et al. (2009) reported that spaceflight led to changes in DNA methylation and gene expression, and some changes can be passed on to offspring, causing transgenic effects. Chen and Wang (2016) used the method of proteomics to explain the change mechanism of rice photosynthesis after spaceflight. In the “Chinese 20th recoverable satellite mission,” we carried two rice varieties DN423 and DN416 for an 18-day flight on low earth orbit (Wei et al., 2006), after returning to the ground, they were planted and analyzed for phenotypic variation, cytology, and genome mutation characteristics (Wei et al., 2006). It was found that DN423 was sensitive to spaceflight, while DN416 was insensitive to spaceflight (Sun et al., 2019), and short-term near-earth flight could induce biological effects of the two varieties of rice. In the SJ-10 mission, we found that the two rice varieties (Dong nong 423 and Dong nong 416) had different responses to the spaceflight in different stages of rice growth through proteomics (Deyong et al., 2020; Zeng et al., 2020). After spaceflight, the plant height of Dongnong 423 (DN3) of rice at the three-leaf stage was significantly higher than that of the control group, while Dongnong 416 (DN6) showed the opposite trend. However, the plant heights of these two types of rice at the tillering stage were not obviously different from those of the control group. In addition, our research results also showed that spaceflight has caused differential expression of proteins involved in energy metabolism, signal transduction, gene transcription, protein synthesis, and protein modification in rice, and we believed that these changes may be induced by reactive oxygen species (ROS) (Deyong et al., 2020; Zeng et al., 2020). Our previous research also found changes in photosynthesis related processes (Cui et al., 2019). The current research on rice after spaceflight was based on genetic material, protein and phenotype. The complete study from the perspective of metabolomics has rarely been reported. As we all knew that there were certain differences between the gene level and the protein level and the final metabolic level. Changes in the gene level and protein level cannot fully reflect the mechanism of rice response to the spaceflight. Therefore, use of metabolomics to study the changes in metabolites of rice in different growth and development stages was particularly important to reveal the response mechanism of rice seeds to spaceflight.

To clarify the complex process responsible for the defense mechanism of crops against the spaceflight at the metabolome level was extremely important for improving the revealing of the mechanism of space biological effects. In this study, we carried two different rice cultivars (Dongnong423 and Dongnong416) in space for 12.5 days through the practice of returning to the SJ-10 satellite. We used metabolomics methods to characterize the changes in the metabolites and basic regulatory pathways of two rice cultivars at different growth and development stages, and verified them with real-time fluorescent quantitative PCR. This work provided novel insights on how rice responded to the spaceflight system at the metabolite level, further provided biomarkers of the impact of spaceflight on organisms at the metabolite level.

## Materials and Methods

### Rice Materials and Spaceflight Conditions

Seeds of two rice cultivars, Dongnong423 (DN3) and Dongnong416 (DN6) were provided by the Agricultural College of Northeast Agricultural University (Harbin, China). The spaceflight conditions described by Deyong et al. (2020). Simply, two varieties of rice dry seeds (DN3 and DN6) were carried on the SJ-10 retrievable satellite for 12.5 days for spaceflight treatment. The total radiation dose was 0.970 mGy (radiation equivalent = 160 μSv/d), and the gravity during flight was 10^–4^ –10^–6^ g. Rice seeds were fixed in the interlayer of the biological irradiation box (Sun et al., 2019), and without mechanical damage such as extrusion and collision. After returning to the ground, the seeds processed for spaceflight (SP3, SP6) and the control seeds (CK3, CK6) were planted in Wuchang City (Harbin, China). The control group was stored on the ground and was not affected by any radiation and microgravity.

Next, we randomly collected the leaves of the two rice cultivars at the three-leaf stage (TLP) and the tillering stage (TS), and stored them at −80°C for further analysis. Therefore, we had 8 groups of rice materials for experiment, namely DN3 rice spaceflight treatment group at three-leaf stage (TLP-SP3); DN3 rice control group at three-leaf stage (TLP-CK3); DN6 rice spaceflight treatment group at three-leaf stage (TLP-SP6); DN6 rice control group at three-leaf stage (TLP-CK6); DN3 rice spaceflight treatment group at tillering stage (TS-SP3); DN3 rice control group at tillering stage (TS-CK3); DN6 rice spaceflight treatment group at tillering stage (TS-SP6); DN6 rice control group at tillering stage (TS-CK6); Five plants were randomly selected and their leaves were mixed to obtain a single biological replicate. In the metabolomics experiment we performed 6 biological replicates, while in other experiments we performed 3 biological replicates.

### Plant Weight Measurement

At the TLP, the aboveground parts of 10 samples of two rice lines were taken to measure the plant weight and calculate the average plant weight. At the TS, the above-ground parts of three samples were taken from each rice line to measure the plant weight and calculate the average plant weight. All operations were repeated three times.

### Detection of Electrolyte Leakage Rate

We used deionized water to wash the residues on the rice leaves, and then measured the electrolyte leakage rate (EL) according to the method described by Lutts et al. (1996). In short, put 0.5 g of leaves into a 25 mL test tube of deionized water, put it at 25°C for 3 h and then measured the conductivity, which was recorded as M1. Next, boil the water bath for 10 min, cool to 25°C, measure the conductivity, and record it as M2. The EL was calculated according to the following formula:

EL = M1/M2 × 100%

### Analysis of Peroxidase, Catalase Activity

Take 0.5 g of the sample into a glass homogenization tube, add 5 mL of phosphate buffer (0.1 mol/L, pH 7.4), and grind evenly in an ice bath. Centrifuge at 11,000 g for 15 min at 4°C. Then collect the supernatant and use it as crude enzyme extract for subsequent enzyme activity analysis. The activity of peroxidase (POD) activity was analyzed according to the method of Castillo et al. (1984). The activity of catalase (CAT) activity was analyzed according to the method of Aebi (1984).

### Detection of Soluble Sugar Content

The soluble sugar content (SSC) was determined according to the anthrone colorimetry method. Take 0.1 g of the sample into a centrifuge tube, add 5 mL of 80% ethanol and extract in a water bath (80°C, 30 min), then centrifuge (6,000 g, 5 min) and collect the supernatant. Repeat the extraction 3 times. Take the supernatant extract and add anthrone reagent, and measure the absorbance at 620 nm after 20 min of color development.

### Extraction of Metabolites and Metabolomics Analysis

The method of metabolomic analyses of the two rice cultivars was modified according to De Vos et al. (2007). In brief, separately take 0.5 g samples after grinding with liquid nitrogen and methanol: acetonitrile: water (1 mL, 2:2:1, v/v/v) and mix them thoroughly, then perform 4°C ultrasonic extraction (100 W, 60 min). After standing for 60 min (−20°C), centrifuge (4°C, 14,000 g, 20 min), and take the supernatant and vacuum dry. Next, add 100 μL of acetonitrile: aqueous solution (1:1, v/v) to the dried sample for reconstitution, and centrifuge (4°C, 14,000 g, 15 min) to collect the supernatant. Acquity UHPLC system (Waters Corporation, Milford, United States) coupled with an AB Sciex Triple TOF 5600 System (AB Sciex, Framingham, MA). Immediately use high performance liquid chromatography (Agilent 1290, United States) tandem Triple TOF 6600 (AB SCIEX, United States) mass spectrometry for analysis. Data acquisition was conducted in full-scan mode (the m/z ranged from 70 to 1,000) in combination with information-dependent acquisition mode. The parameters were set as follows: ion spray voltage, 5,500 V (+) and 5,500 V (–); ion source temperature, 600°C (+) and 600°C (–); collision energy, 35 ± 15 eV (–); curtain gas of 30 PSI; The original data was converted into.mzML format by ProteoWizard, and then the XCMS program was used for peak alignment, retention time correction and peak area extraction. Metabolite structure identification is carried out by accurate mass matching (<25 ppm) and secondary spectrum matching. Reference material databases built by Dalian Institute of Chemical Physics and Shanghai Zhongke New Life Biotechnology Co., Ltd. After XCMS extracts the data, metabolites with more than 50% missing values in the group will be removed and will not participate in subsequent analysis. At the same time, delete extreme values and normalize the total peak area of the data to ensure parallel comparison between samples and metabolites. The data was input into the software SIMCA-P 14.1 (Umetrics, Umea, Sweden) for pattern recognition, preprocessed by Pareto-scaling, and then subjected to multi-dimensional statistical analysis. Partial least squares discriminant analysis (PLS-DA) and metabolic pathway analysis both use MetaboAnalyst 4.0 software^1^. Principal component analysis (PCA) uses R software.

### Total RNA Extraction and Real-Time PCR

Same as previous research Deyong et al. (2020). Use TaKaRa kit (9767) to extract total RNA from rice leaves, Simply, rice leaves were quickly ground into powder in liquid nitrogen, and total RNA is extracted according to the instructions of TaKaRa kit (9767). 1% (w/v) denaturing agarose gel electrophoresis and Micro Drop (BIO-DL Co. Ltd, Shanghai, China) were used to evaluate RNA quality and total RAN concentration, respectively. Then use TaKaRa kit (RR073A) to reverse transcribed RNA into cDNA, and stored at −20°C. SYBR Premix Ex Taq II [TaKaRa kit (820A)] was used to detect gene expression. Relative expression levels of genes were determined using a relative quantitative method (2–ΔΔCT) (Cui et al., 2019). All primers are listed in Supplementary Table 1.

## Results

### The Change of Morphological and Physiological Traits in Two Cultivars

**FIGURE 1:**
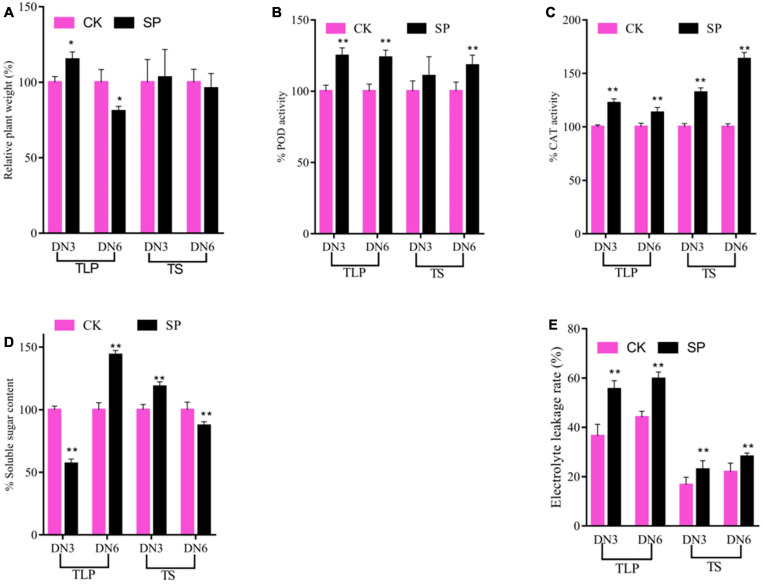
The morphological and physiological changes of rice induced by spaceflight. **(A)** Percent values of plane weight. **(B)** Percent values of POD activity. **(C)** Percent values of CAT activity. **(D)** Percent values of soluble sugar content. **(E)** The electrolyte leakage rate change. TLP, three-leaf stage; TS, tillering stage; Pink column represents the control group; black column represents spaceflight group. Data are expressed as mean ± SD, *n* = 9, * and ** indicate significant difference at *p* < 0.05 and *p* < 0.01 by Student’s *t*-test (GraphPad Prism 7), respectively.

In order to better understand the differences in the response of different rice strains to spaceflight at different growth and development stages, we analyzed the plant weight of rice and some physiological indicators of response to abiotic stress. Compared with the control group, the plant weight of the two rice cultivars changed during the TLP, while no difference at the TS (Figure 1A). Interestingly, the plant weight of the DN3 spaceflight group was significantly higher than that of the control group at the TLP, while the plant weight of DN6 was significantly lower than that of the control group at this stage (Figure 1A). We measured antioxidant enzyme activities, SSC and EL to evaluate the similarities and differences of the response of two different rice cultivars to the spaceflight (Figures 1B–E). In TLP and TS, the POD activity, CAT activity and EI in the two rice cultivars leaves treated by spaceflight were higher than the control (Figures 1B,C,E). In detail, in SP3, the activity of POD was significantly increased by 24.83% during TLP, while the activity of POD during TS increased, but there was no statistical significance. In SP6, the activity of POD increased by 23.67 and 18.08% in TLP and TS, respectively (Figure 1B). The activity of CAT increased by 22.3 and 32.2% in TLP and TS of SP3, respectively, and increased by 13.2 and 63.5% in TLP and TS of SP6, respectively (Figure 1C). In the two different growth stages of SP3 and SP6, EI increased by 51.9, 35.5, 37.4, and 28.2%, respectively (Figure 1C). We also characterized the changes of SSC in two rice cultivars leaves. The content of SSC was decrease in TLP-SP3 and increase in TS-SP3 (Figure 1D). However, we saw the opposite result in SP6 with SP3 (Figure 1D). Based on the above results, it can be preliminarily determined that the spaceflight has caused changes in the physiological state of rice, and there were certain differences in the response of the two different rice lines to the spaceflight.

### Spaceflight Treatment Globally Reversed the Metabolic Profiles in Rice

**FIGURE 2:**
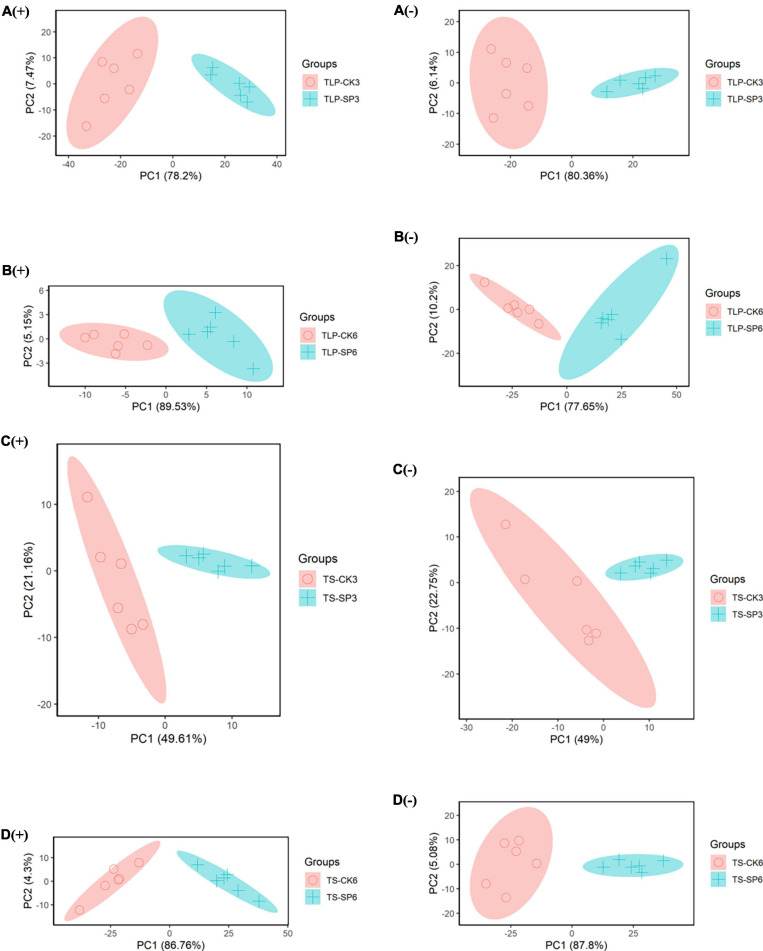
PCA score map of different rice sample groups based on UHPLC-QTOF/MS data. **(A)** Rice samples of DN3 at the TLP, **(B)** rice samples of DN6 at the TLP, **(C)** rice samples of DN3 at the TS, **(D)** rice samples of DN6 at the TS; (+) and (–) represent positive and negative ion; TLP and TS represent three leaf period and tillering stage.

Metabolomics analysis was used to further reveal the impact of spaceflight on two different strains of rice. In the positive ion or negative ion mode, a clear separation between the CK group and the SP group was observed in the PCA mode of rice samples at different periods (Figures 2A–D), which indicated that the spaceflight treatment can effectively change the metabolic characteristics of rice.

**FIGURE 3:**
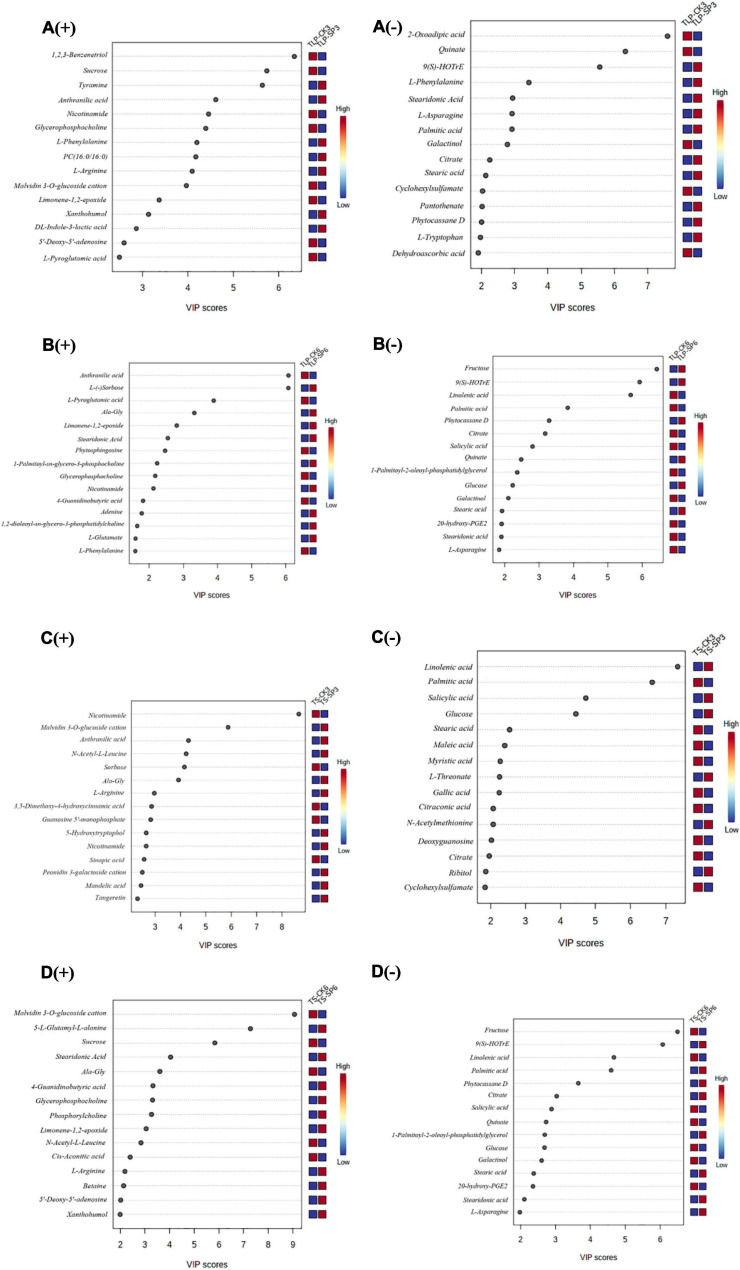
Variable importance in projection (VIP) scores of the top 15 metabolites in grains of the two rice cultivars in different growth stage. Results were from the partial least squares-discriminant analysis (PLS-DA). **(A)** Rice samples of DN3 at the TLP, **(B)** rice samples of DN6 at the TLP, **(C)** rice samples of DN3 at the TS, **(D)** rice samples of DN6 at the TS; (+) and (–) represent positive and negative ion; TLP and TS represent three leaf period and tillering stage. The higher the VIP scores, the larger the contributions. The relative contents of metabolites were displayed by the color boxes, with red indicating high and green indicating low contents.

In order to explore the main reasons for the differences in rice metabolites of different rice at different growth stages, we conducted partial least square discriminant analysis (PLS-DA). PLS-DA recognized 15 main components responsible for separation based on their VIP scores (Figure 3). These 15 metabolites revealed the main reasons for the separation of metabolites from each other in Figure 3. Obviously, 123-Benzenetriol, sucrose, tyramine, 2-oxoadipic, quinate, and 9(S)-HOTrE have the most significant changes in the TLP stage of SP3. In the TS stage, nicotinamide, malvidin 3-o-glucoside cation, anthranilic acid, linolenic acid, and palmitic acid had the most significant changes (Figures 3A,C). The PLS-DA and VIP scores identified L-sorbose, fructose, L-pyroglutamic, 9(S)-HOTrE, malvidin 3-o-glucoside cation, anthranilic acid, linolenic acid, and palmitic acid as dominant components for separation between the CK and SP6 (Figures 3B,D).

### Spaceflight Treatment Altered the Metabolite Profiles in Different Biological Samples of Rice

**FIGURE 4:**
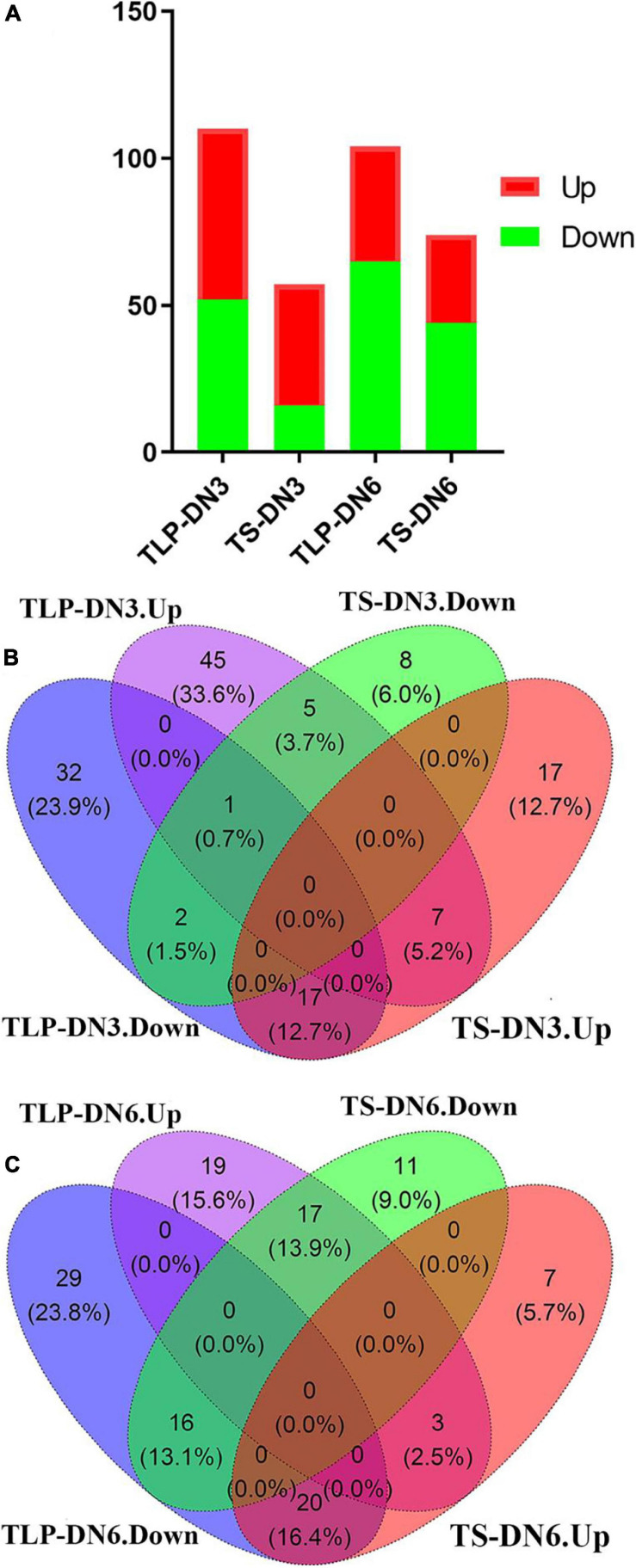
The number of up-regulated and down-regulated DEMs in two different growth stages **(A)**. Venn diagram showed the overlaps of the DEMs in DN3 **(B)**. Venn diagram showed the overlaps of the DEMs in DN6 **(C)**.

**FIGURE 5:**
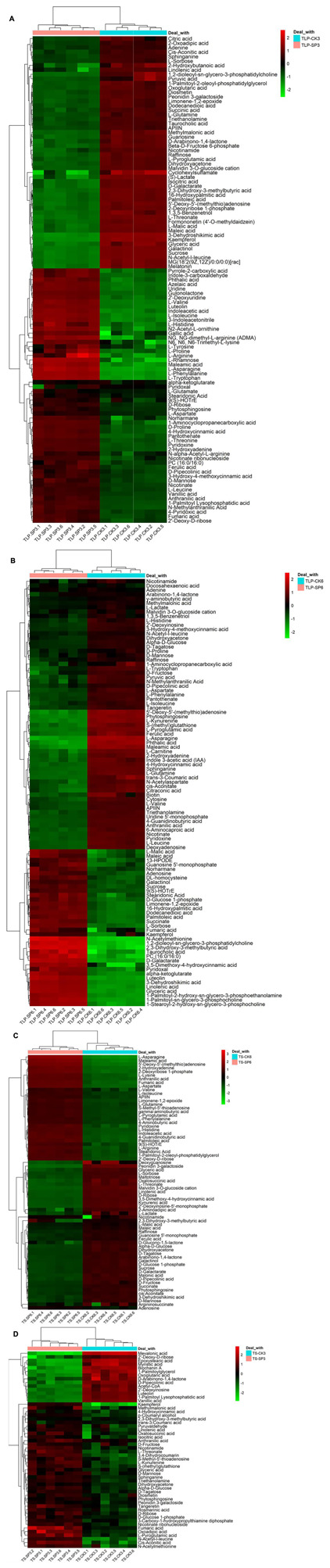
Hierarchical cluster heat map of different metabolites under spaceflight stress of rice leaves; **(A)** Rice samples of DN3 at the TLP, **(B)** rice samples of DN6 at the TLP, **(C)** rice samples of DN3 at the TS, **(D)** rice samples of DN6 at the TS.

To understand the differences in the response of the two rice varieties to the spaceflight during different growth periods, we comprehensively considered the Fold change (FC), *P*-value and VIP score. We defined metabolites with FC ≥ 1.2 or FC ≤ 0.83, *P* ≤ 0.05 and VIP score ≥ 1 as differential metabolites (DEMs). The concentrations of 110 (SP VS CK) metabolites in TLP-DN3 (Supplementary Table 2) and 57 (SP VS CK) metabolites in TS-DN3 (Supplementary Table 3) varied significantly after the spaceflight (Figure 4A). In TLP-DN6 and TS-DN6, there were 104 (SP VS CK) (Supplementary Table 4) and 74 (SP VS CK) metabolites (Supplementary Table 5) that changed significantly (Figure 4A). In the DN3, 32 metabolites (23.9%) changed significantly in both TLP and TS stages (Supplementary Figure 1A). However, only 10 metabolites (7.4%) among the 33 common metabolites maintained the same trend of changes in the two growth and development stages (Figure 4B). Fifty six metabolites (45.9%) changed significantly in the TLP and TS stages of DN6 (Supplementary Figure 1B). There were 19 metabolites (15.6%) that maintained the same trend of change in the two growth stages of DN6 (Figure 4C). In TLP-SP3, the concentration of L-Asparagine was increased by 38.93-fold, and the concentration of Kaempferol decreased by 91% (Supplementary Table 2). The concentration of most metabolites in TS-SP3 increased significantly. What’s more interesting was that most of the amino acids in TLP-SP3 have changed significantly but there was no change in TS-SP3 (Supplementary Table 3). The concentration of most metabolites in TLP-SP6 and TS-SP6 decreased, and the concentration of L-Asparagine and Maleamic acid decreased the most in TLP-SP6, reducing 98 and 96%, respectively (Supplementary Table 4). In addition, the number of differential metabolites at TS stage in DN3 and DN6 were fewer than that of TLP stage, which may imply that with the growth and development of rice, the influence of spaceflight on it was weakening. In order to further analyze the response patterns of the two rice varieties to the spaceflight at different growth and development stages, we conducted a hierarchical cluster analysis of all the different metabolites in different groups (Figure 5). Our results showed that the spaceflight group was significantly separated from the control group (Figure 5), which indicated that the spaceflight had an impact on both types of rice. However, the expression patterns of metabolites in different growth stages of the two rice were different, which suggested that there were differences in the response patterns of the two rice varieties to the spaceflight.

### Cluster Analysis of Spaceflight Induced Changes in Metabolites

**FIGURE 6:**
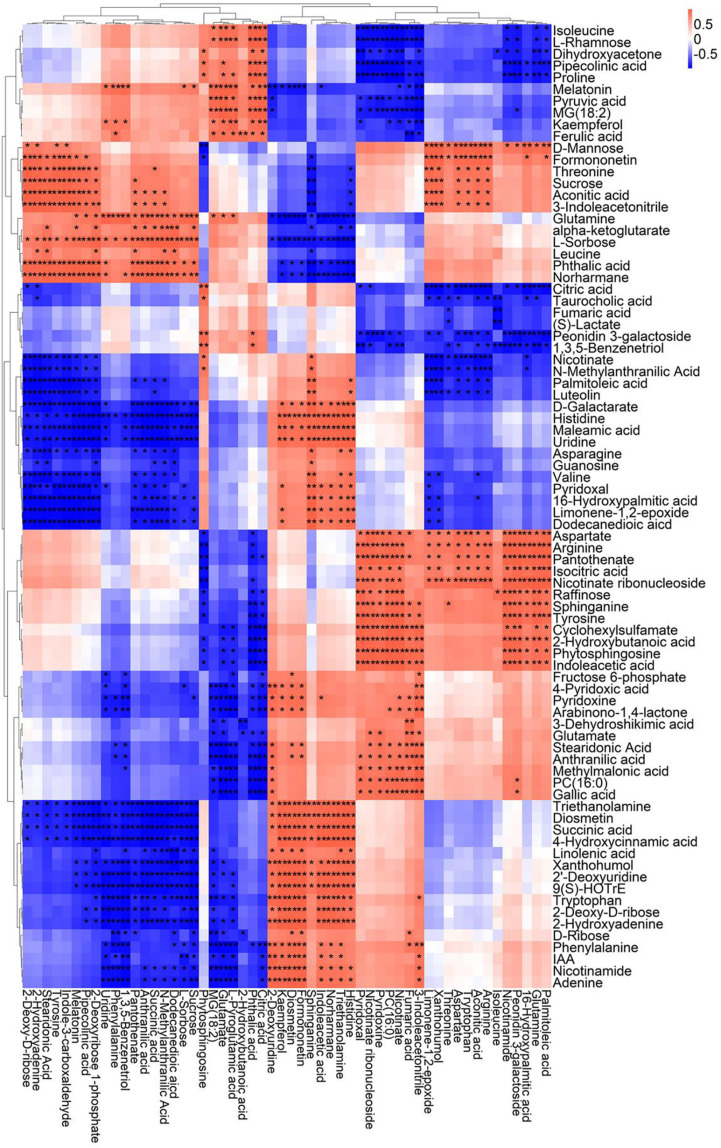
Correlation analysis of TLP-DN3 leaf metabolites subjected to spaceflight treatment. A correlation matrix was generated using Pearson’s correlation on six biological replicates of CK and SP-treated plants. Red color shows positive correlation whereas blue color represents negative correlation among the metabolites under spaceflight treatment. ** and * indicate significant difference at *p* < 0.01 and *p* < 0.05 by Pearson’s correlation test, respectively.

**FIGURE 7:**
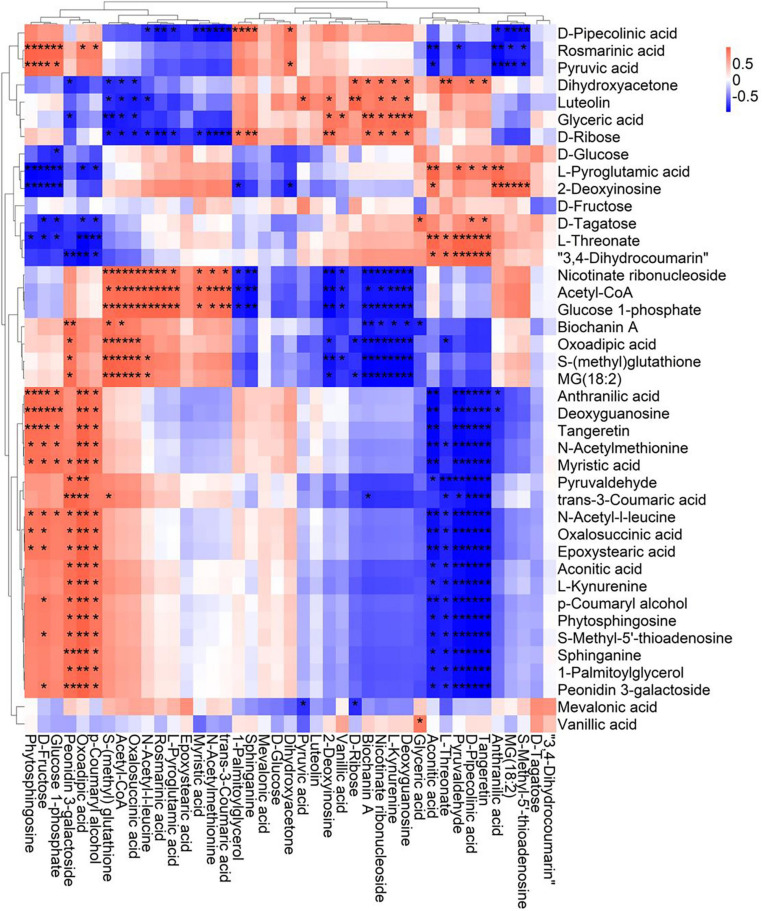
Correlation analysis of TS-DN3 leaf metabolites subjected to spaceflight treatment. A correlation matrix was generated using Pearson’s correlation on six biological replicates of CK and SP-treated plants. Red color shows positive correlation whereas blue color represents negative correlation among the metabolites under spaceflight treatment. ** and * indicate significant difference at *p* < 0.01 and *p* < 0.05 by Pearson’s correlation test, respectively.

**FIGURE 8:**
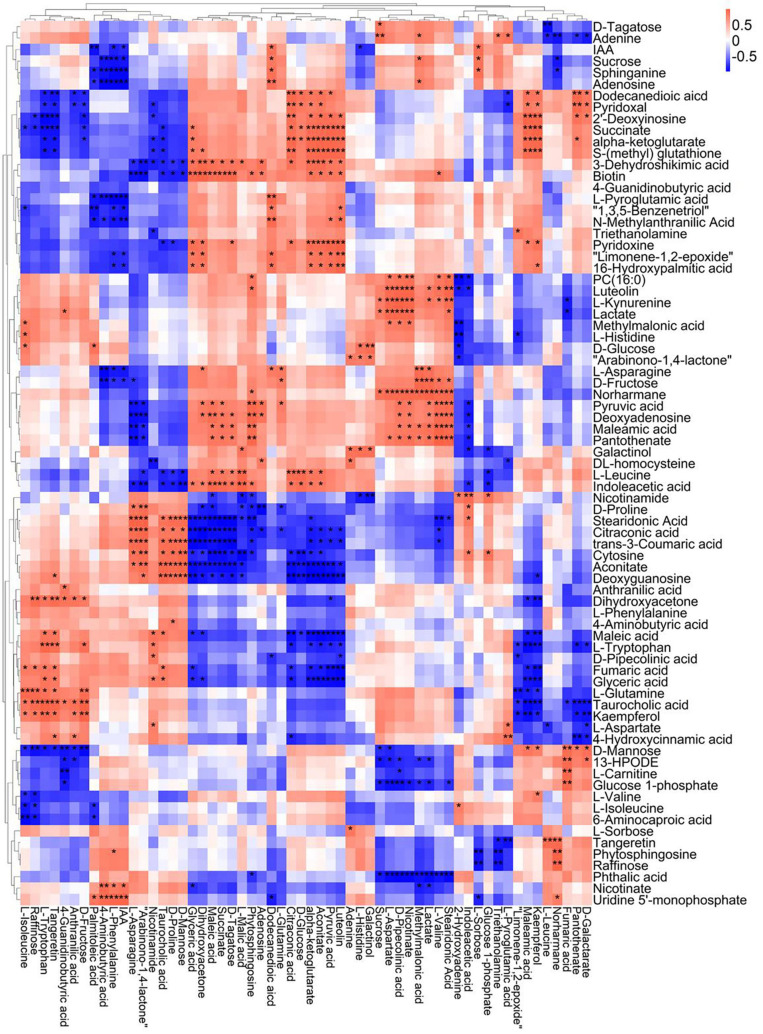
Correlation analysis of TLP-DN6 leaf metabolites subjected to spaceflight treatment. A correlation matrix was generated using Pearson’s correlation on six biological replicates of CK and SP-treated plants. Red color shows positive correlation whereas blue color represents negative correlation among the metabolites under spaceflight treatment. ** and * indicate significant difference at *p* < 0.01 and *p* < 0.05 by Pearson’s correlation test, respectively.

**FIGURE 9:**
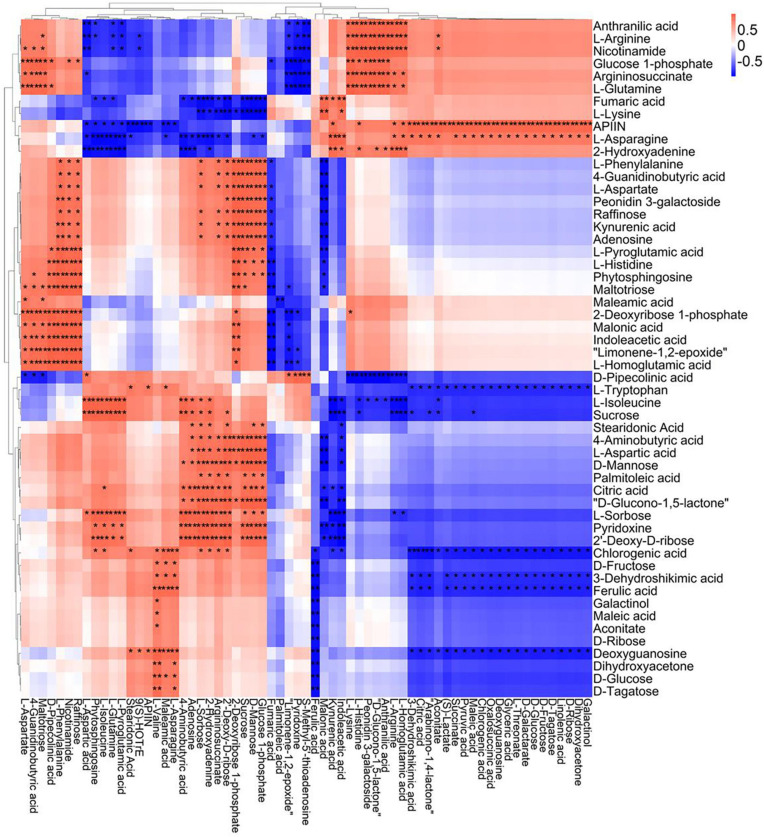
Correlation analysis of TS-DN6 leaf metabolites subjected to spaceflight treatment. A correlation matrix was generated using Pearson’s correlation on six biological replicates of CK and SP-treated plants. Red color shows positive correlation whereas blue color represents negative correlation among the metabolites under spaceflight treatment. ** and * indicate significant difference at *p* < 0.01 and *p* < 0.05 by Pearson’s correlation test, respectively.

A correlation matrix was generated using Pearson correlation test to calculate the correlation coefficient between metabolites in order to study the interdependence of different metabolites. Correlation matrix revealed the relationship between different metabolites (Figures 6–9). Compared with TLP-CK3, TLP-SP3 except for aconitic acid and succinic acid, most of the organic acids (fumaric acid, citric acid, alpha-ketoglutarate) that involved in energy metabolism exhibited a negative correlation with amino acids (isoleucine, histidine, arginine, aspartate, glutamine, threonine, phenylalanine, tyrosine) (Figure 6). It was worth mentioning that aconitic acid showed a positive correlation with arginine, aspartate, and tyrosine, but a negative correlation with histidine (Figure 6). In addition, we noticed that succinic acid was positively correlated with histidine, and negatively correlated with phenylalanine and tyrosine (Figure 6). The results in Figure 6 also showed a positive correlation between changes in sugars (sucrose, mannose, raffinose) and most metabolites (Figure 6). Compared with TS-CK3, pyruvic acid was negatively correlated with aconitic acid in TS-SP3, and it was positively correlated with fructose (Figure 7). Glucose and tagatose were negatively correlated with glucose 1-phosphate, and fructose (Figure 7). In addition, we haven’t found any relevant changes in amino acid metabolism in Figure 7. This showed that after spaceflight, DN3 had different metabolite profiles at different developmental stages. Similarly, we also analyzed the correlation between the metabolites in the two growth stages of DN6 (Figures 8, 9). Compared with TLP-CK6, pyruvic acid in TLP-SP6 was positively correlated with maleic acid, succinate, glutamine, valine, stearidonic acid, nicotinate, adenosine, and lactate (Figure 8). Moreover, our results showed that most of the amino acids (aspartate, glutamine, proline, histidine, tryptophan, isoleucine, valine, leucine) showed a negative correlation with galactarate, limonene-1,2-epoxide, maleamic acid, malic acid (Figure 8). Interestingly, the isoleucine in the spaceflight group and the control group showed a negative correlation (Figure 8), which indicated that isoleucine in this period was seriously affected by spaceflight. Glutamine was positively related to aspartate and histidine (Figure 9). There was also a positive correlation between most amino acids and carbohydrates (Figure 9). Our correlation analysis results revealed the metabolic relationship between organic acid metabolism, amino acid metabolism, and carbohydrates.

### Analysis of Metabolic Pathways of Two Rice Cultivars

**FIGURE 10:**
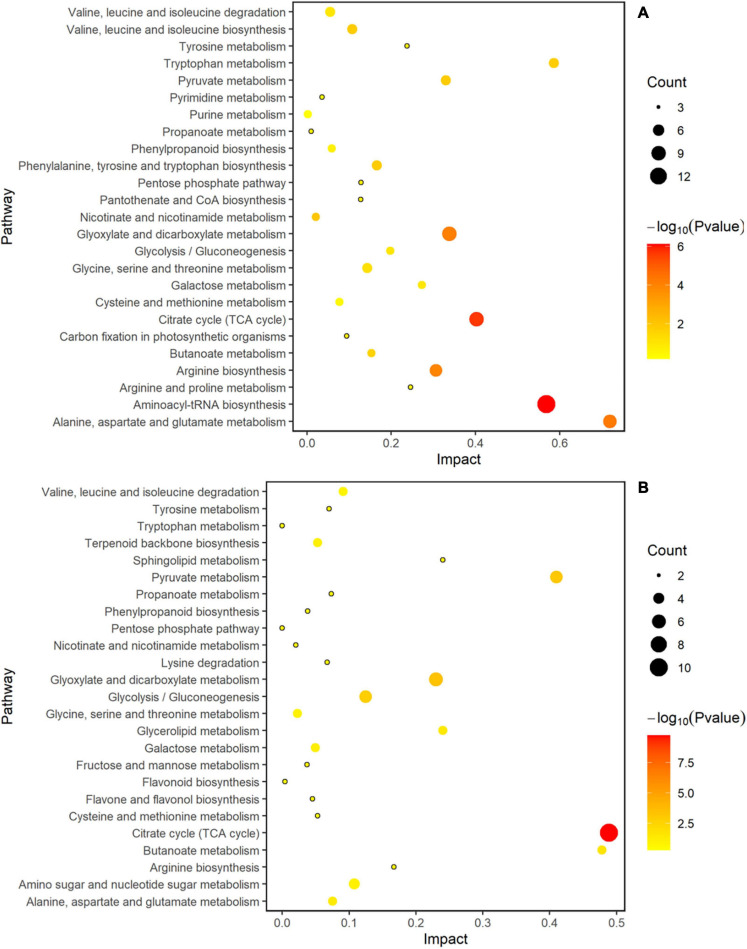
Analysis of metabolic pathways of TLP-DN3 and TS-DN3. Results were from the analyses using the MetaboAnalyst 4.0 software. Every circle represents a metabolic pathway, with red color indicating higher impact and yellow color indicating lower impact. The pathways with *p*-value < 0.05 were determined to have significant changes. The size of the circle represents the number of DEMs participating in the pathway. **(A)** Rice samples of DN3 at the TLP, **(B)** rice samples of DN3 at the TS.

**FIGURE 11:**
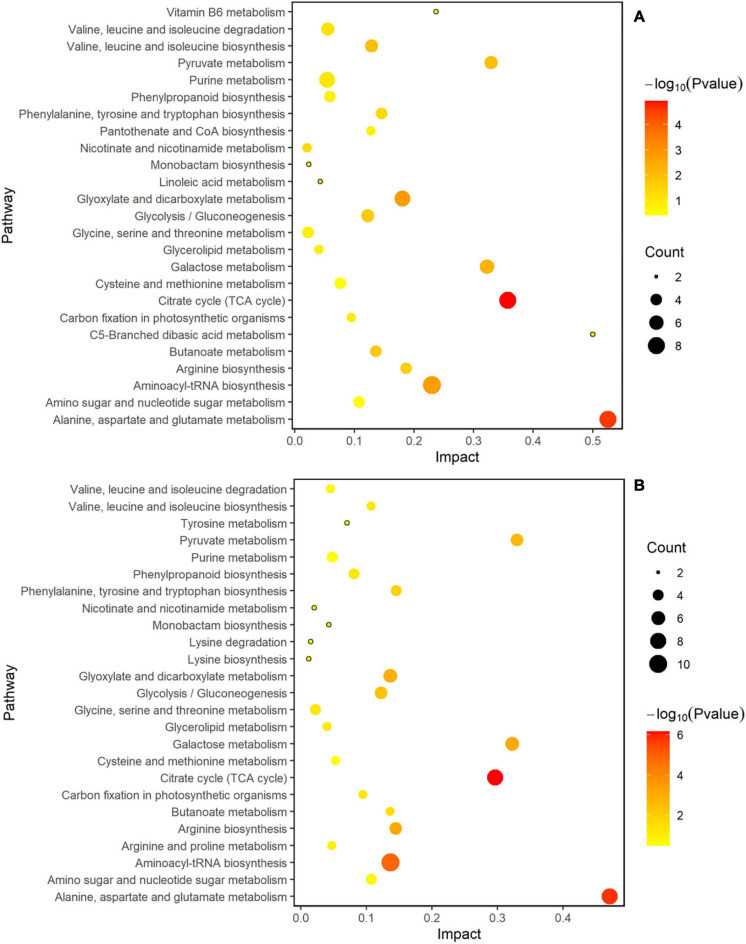
Analysis of metabolic pathways of TLP-DN6 and TS-DN6. Results were from the analyses using the MetaboAnalyst 4.0 software. Every circle represents a metabolic pathway, with red color indicating higher impact and yellow color indicating lower impact. The pathways with *p*-value < 0.05 were determined to have significant changes. The size of the circle represents the number of DEMs participating in the pathway. **(A)** Rice samples of DN6 at the TLP, **(B)** rice samples of DN6 at the TS.

MetaboAnalyst 4.0 software was used to analyze the metabolic pathways to better understand the impact of spaceflight on the biological processes of two rice cultivars, and the analysis results were displayed by bubble charts. The involved pathways were revealed in Figures 10, 11. Thirteen and eight metabolic pathways were significantly enriched in TLP-SP3 and TS-SP3, respectively (Figures 10A,B). These pathways mainly involved amino acid metabolism, TCA cycle, and pyruvate metabolism. There were 14 pathways that were significantly enriched in TLP-SP6 (Figure 10A). They were: (1) TCA cycle; (2) Alanine, aspartate and glutamate metabolism; (3) Glyoxylate and dicarboxylate metabolism; (4) Aminoacyl-tRNA biosynthesis; (5) Galactose metabolism; (6) Valine, leucine and isoleucine biosynthesis; (7) Pyruvate metabolism; (8) Butanoate metabolism; (9) Glycolysis/Gluconeogenesis; (10) Linoleic acid metabolism; (11) Arginine biosynthesis; (12) Nicotinate and nicotinamide metabolism; (13) Phenylalanine, tyrosine and tryptophan biosynthesis; (14) C5-Branched dibasic acid metabolism (Figure 7). However, there were only 11 significant changes in TS-SP6 (Figure 6B). They were (1) TCA cycle; (2) Alanine, aspartate and glutamate metabolism; (3) Aminoacyl-tRNA biosynthesis; (4) Arginine biosynthesis; (5) Galactose metabolism; (6) Glyoxylate and dicarboxylate metabolism; (7) Pyruvate metabolism; (8) Glycolysis/Gluconeogenesis; (9) Phenylalanine, tyrosine biosynthesis, tyrosine, and try metabolism; (11) Monobactam biosynthesis. Among them, amino acid metabolism, TCA cycle, glycolysis/gluconeogenesis, pyruvate metabolism, glyoxylate and dicarboxylate metabolism, galactose metabolism, aminoacyl-tRNA biosynthesis were all altered significantly for both growth stage (Figure 10). It is worth noting that pyruvate metabolism, TCA cycle, and amino acid metabolism were significantly enriched in the different growth and development stages of the two rice cultivars (Figures 10, 11).

### Expression of Genes Related to Metabolic Pathways

**FIGURE 12:**
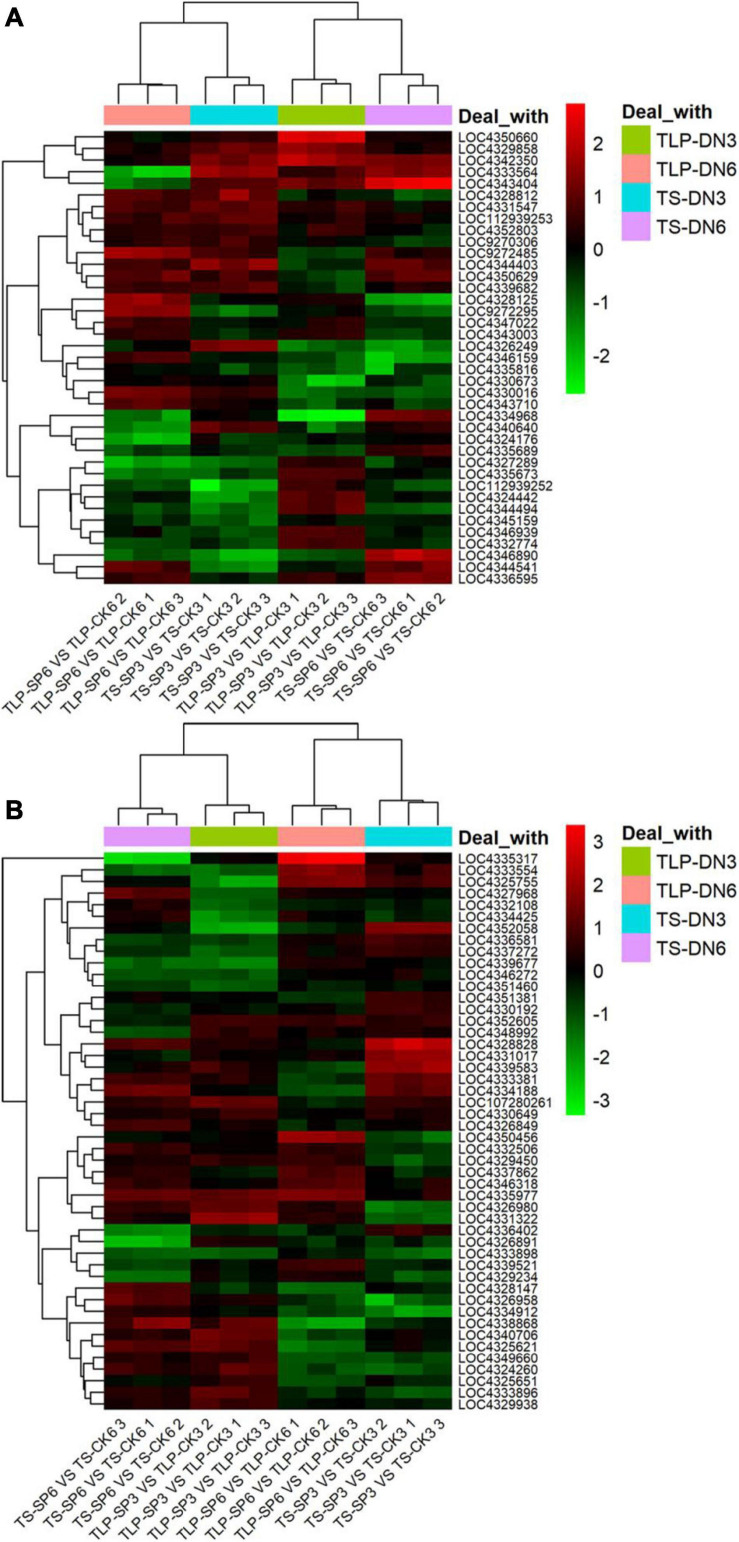
The relative expression levels of TCA cycle and amino acid metabolism genes in two different rice varieties. The heat map shows the expression fold change in rice leaves obtained by qRT-PCR. The relative expression is calculated by the gene expression of the space group and the control group at different growth periods. **(A)** The expression of genes related to the TCA cycle. **(B)** Expression of genes related to amino acid metabolism. Red indicates that the gene expression is up-regulated, and green metabolism is down-regulated. The normalization of gene expression was obtained by log2 transformation. Hierarchical clustering analysis was performed using R.

We noticed that two kinds of rice amino acid metabolism and TCA cycle were reshaped by the spaceflight. It was well known that the TCA cycle was significantly changed under stress to regulate the adaptability of plants to stress, and amino acids can be converted into intermediate products to compensate for the level of intermediate metabolites in TCA cycle. In addition, changes in the TCA cycle indicated that mitochondrial function was affected. Therefore, we explored the changes in genes related to the TCA cycle and amino acid metabolism (Figure 12 and Supplementary Table 6). The expression of genes related to the TCA cycle showed different expression patterns at different growth stages of the two rice varieties. In SP3, the expression of most TCA cycle-related genes was down-regulated during TLP and up-regulated during TS (Figure 11A and Supplementary Table 6). SP6 showed the opposite trend with SP3. In SP6, the expression of TCA cycle-related genes was up-regulated during TLP, and down-regulated during TS (Figure 12A and Supplementary Table 6). The expression of genes related to amino acid metabolism also showed different expression patterns in different growth stages of the two rice varieties. In SP3, the expression of genes related to amino acid metabolism was up-regulated in TLP, and down-regulated in TS (Figure 12B and Supplementary Table 6). In SP6, the expression of genes related to amino acid metabolism during TLP was down-regulated, and the expression of TS was up-regulated (Figure 12B and Supplementary Table 6).

## Discussion

Our team has conducted simulated radiation studies with different doses on the ground in the early stage (Wei et al., 2006; Shi et al., 2014), and found that even if there is only 2 mGy during spaceflight, the mutation rate caused is higher than that caused by 2 Gy on the ground (Shi et al., 2014). Therefore, the space environment is a special abiotic stress environment. In recent years, various omics methods have been used to study the effects of spaceflight on plants, but the application of metabolomics has not been reported. Our research reported the effects of spaceflight on the proteome of two different rice varieties (DN6 and DN3) (Deyong et al., 2020; Zeng et al., 2020), and found that there were certain differences in their response to spaceflight. Here, we compared the changes in the metabolome profiles of two rice lines at different growth and development stages to reveal their metabolic responses to the spaceflight, and to study the chemical differences and conservation between varieties.

### The Effect of Spaceflight on the Overall Morphology and Physiology of Two Rice Varieties

In TLP, the plant weights of the two rice varieties showed different trends in the spaceflight group and the control group (Figure 1A), which was the same as the trend of rice plant height changes we previously reported (Deyong et al., 2020; Zeng et al., 2020). As we all know, the changes in antioxidant enzyme activity reflected the biological redox state in plants (Mittler, 2017). Our results showed that the activities of POD and CAT in rice were increased in spaceflight group (Figures 1B,C), which indicated that the redox state in rice was disturbed at this time. Soluble sugar played an obvious central role in plant structure and metabolism (Couée et al., 2006). Moreover, the sugar signal produced by soluble sugar was connected in series with the ROS signal to control the redox state in the body (Contento et al., 2004; Loreti et al., 2005). The content of soluble sugar changed during the TLP and TS of two different rice varieties, and the changing trends were different (Figure 1D). This indicated that rice may adjust to sugar metabolism to cope with the effects of spaceflight, but there may be some differences in the mechanism. Electrolyte leakage rates was a sign of stress response in plant cells (Demidchik et al., 2014). Increases in electrolyte leakage rates have been reported in abiotic stresses such as heavy metals (Murphy and Taiz, 1997; Demidchik et al., 2003), drought (Shcherbakova and Kacperska, 1983), waterlogging (Shabala, 2011), heat (Liu and Huang, 2000), and oxidative stress (Demidchik et al., 2010). This study obtained the same changes as previously reported. The SP group of electrolyte leakage rates was higher than that of the CK group in the two types of rice (Figure 1E). Taken together, our results demonstrated that spaceflight had a stress response to two different varieties of rice, and their response mechanisms to space stress may be different.

### Spaceflight Changes the Process of TCA

**FIGURE 13:**
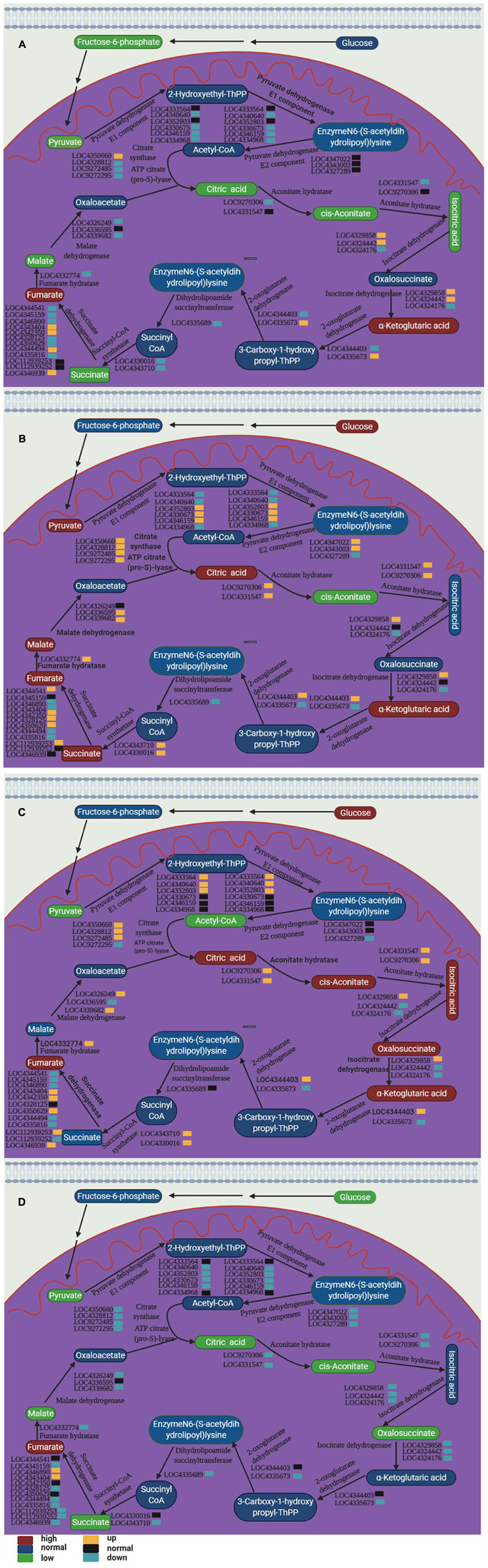
TCA cycle pathways of two rice varieties. After spaceflight, the two rice varieties developed into TLP and TS respectively. TLP-DN3 **(A)**, TLP-DN6 **(B)**, TS-DN3 **(C)**, and TS-DN6 **(D)** show changes in the expression levels of metabolites and genes mapped to the TCA cycle pathway. The relative content of each metabolite in the grain is displayed in the form of a heat map from low (green) to high (red), as shown by the color scale. Enzymes related to these pathways are marked in red, and genes encoding enzymes are placed next to them. Similarly, gene expression levels are represented by yellow (up-regulated) and cyan (down-regulated). Blue and black, respectively, represent no significant changes in metabolites and gene expression.

The changes in plant mitochondrial function caused by spaceflight have been confirmed in previous studies (Sugimoto et al., 2014). In this study, some intermediate products involved in the TCA have undergone significant changes (Supplementary Tables 2–5). This indicated that spaceflight may have an impact on rice mitochondria, which further confirmed the conclusion of the changes in mitochondrial function shown in our proteomics results (Deyong et al., 2020; Zeng et al., 2020). In order to further confirm the influence of spaceflight on the TCA cycle of rice, we integrated the results of metabolomics and qRT-PCR to draw the TCA cycle metabolic pathway diagram (Figure 13).

The results of the transcriptome have confirmed that the spaceflight has induced changes in the *Arabidopsis* TCA cycle-related genes (Sugimoto et al., 2016), but the changes in the metabolic level of TCA were reported for the first time in this study. Under many abiotic stresses, TCA flux would change significantly (Chen et al., 2019). Therefore, TCA was generally considered to be an important physiological response that regulated the adaptability of plants to abiotic stress. In addition, the intermediate metabolites of the TCA cycle were precursors for the synthesis of amino acids, fatty acids and secondary metabolites (Kumari and Parida, 2018; Chen et al., 2019). In our research, the content of aconitic acid, alpha-ketoglutarate, maleic acid, succinate, fumaric acid, pyruvic acid, citric acid, isocitric acid and other organic acids have changed significantly (Figure 13).

In TLP-SP3 and TS-SP6, the content of most intermediate products in the TCA cycle was reduced, and the expression of enzyme genes encoding the enzymatic reaction in this pathway was down-regulated, which indicated that the TCA cycle rate in TLP-SP3 and TS-SP6 was reduced. However, in TLP-SP6 and TS-SP3, the content of intermediate metabolites increased, and the expression of related genes related to enzymatic reactions was up-regulated, indicating that the TCA rate in TLP-SP6 and TS-SP3 increased. Therefore, the metabolic fluxes of the TCA cycle were different in the two different growth and development stages of the same rice strain (Figure 13). What’s more interesting was that the TCA cycle fluxes of the two types of rice at the same growth and development period were different, and the two types of rice showed opposite trends (Figure 13). Under salt stress, the content of pyruvate, oxalic acid, malic acid, citric acid and succinic acid in *Salvadora persica* decreased (Kumari and Parida, 2018). This decrease may be the decrease of TCA cycle activity and the increase of carbon structure, which was used to synthesize the compounds needed for stress response (Kiani-Pouya et al., 2017). In addition, under the stress of 2,2′,4,4′-Tetrabromodiphenyl ether, the TCA cycle rate of rice increased, for the reason that 2,2′,4,4′-Tetrabromodiphenyl ether would activate a series of reactions to provide energy for the normal life of rice (Chen et al., 2019). Therefore, the effects of stress conditions on the TCA cycle of plants may be different, which showed again in our research that there were differences in the mechanisms of the two types of rice in response to the spaceflight at different growth stages.

Citric acid was an important organic acid that participated in the absorption of iron ions by plants and numerous physiological and biochemical reactions (Hell and Stephan, 2003). It was reported that citric acid involved in aluminum poisoning (Ma and Furukawa, 2003), iron stress (Shlizerman et al., 2007), heavy metal stress tolerance (Gao et al., 2010), salt stress (Sun and Hong, 2011). Therefore, the change in citric acid content in this study reflected the differences in the resistance of the two rice varieties to the spaceflight at different growth stages.

In our results, we also observed a significant increase in alpha-ketoglutarate in TLP-SP3, TLP-SP6, and TS-SP3. Isocitrate dehydrogenase can convert isocitrate into α-ketoglutarate through oxidative decarboxylation, so that plants can produce ATP. The content of isocitrate in TS-SP3 was consistent with the content of alpha-ketoglutarate, which indicated that the increase of alpha-ketoglutarate in TLP-DN3 may be caused by the change of isocitrate. It was worth noting that only one of the three genes encoding isocitrate dehydrogenase was up-regulated, and our proteomic results did not show increased abundance of isocitrate dehydrogenase (Deyong et al., 2020). This indicated that the activity of isocitrate dehydrogenase may be regulated. In addition, we also noticed that fumaric acid was increased in TLP-SP3, TLP-SP6, TS-SP3, and TS-SP6. The increase of fumaric acid helped to improve the tolerance of plants to stress conditions (Song et al., 2012).

Our results revealed that the TCA rates of the two rice varieties at different growth and development stages showed different patterns after spaceflight. This was caused by the different response mechanisms of the two rice varieties seeds to the spaceflight.

### Spaceflight Changes the Process of Amino Acid Metabolism

**FIGURE 14:**
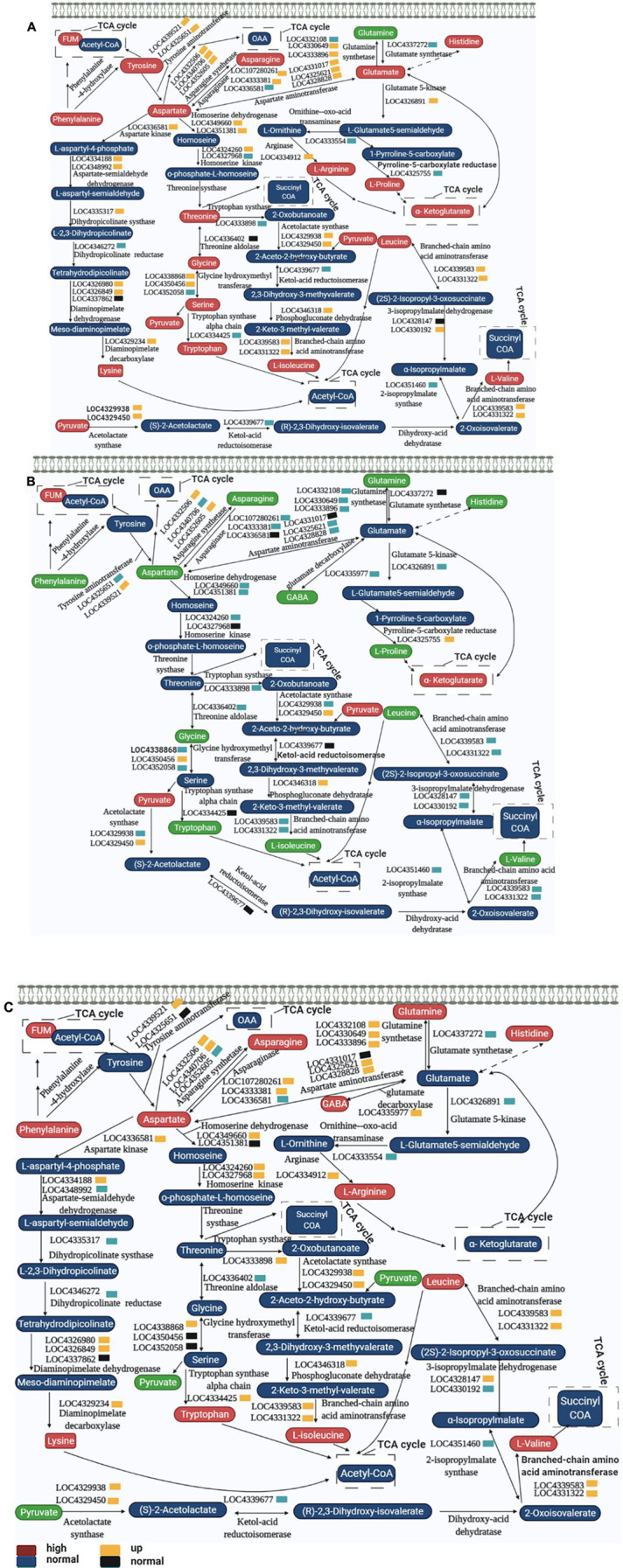
Amino acid metabolism pathways of two rice varieties. After spaceflight, the two rice varieties developed into TLP and TS, respectively. TLP-DN3 **(A)**, TLP-DN6 **(B)**, and TS-DN6 **(C)** show changes in the expression levels of metabolites and genes mapped to amino acid metabolism pathways. The relative content of each metabolite in rice is displayed in the form of a heat map from low (green) to high (red), as shown by the color scale. Enzymes related to these pathways are marked in red, and genes encoding enzymes are placed next to them. Similarly, gene expression levels are represented by yellow (up-regulated) and cyan (down-regulated). Blue and black, respectively represent no significant changes in metabolites and gene expression.

Amino acids played a vital role in plants. They were the basic building blocks of proteins and provide necessary intermediate metabolites for many metabolic reactions. It was an important molecular form of plant organic nitrogen. Therefore, strict control of the biosynthesis, degradation and transportation of amino acids can meet the needs of plants for nitrogen and carbon utilization (Pratelli and Pilot, 2014). Previous studies have shown that spaceflight causes changes in protein during amino acid metabolism (Sun et al., 2019). Here, we combined the results of metabolomics and qRT-PCR to map the complex network of amino acid transformation in rice at different growth and development stages after spaceflight to reveal the changes of amino acid metabolism in rice (Figure 14).

In TLP-SP3, there were 15 kinds of amino acids that showed a complex network relationship. Among these 15 kinds of amino acids, except for the decrease in glutamine content, the others all showed an increasing trend (Figure 14A). The expression of genes encoding these amino acid interconversion enzymes also showed up-regulation (Figure 14A), so we confirmed that amino acid metabolism was activated when DN3 developed into TLP after spaceflight. Interestingly, we did not find significant changes in these amino acids in TS-SP3 (Supplementary Table 3), but qRT-PCR results showed a significant down regulation of the genes encoding the enzyme (Figure 12B). This may also be one of the reasons why the amino acids of TS-SP3 have not changed significantly. For DN6, the amino acid content was reduced during TLP (Figure 14B), and the expression of enzyme genes was mostly down-regulated (Figure 14B), while the content of amino acids increased in TS (Figure 14C), and the expression of enzyme gene was up-regulated (Figure 14C). Therefore, the amino acid metabolism in TLP-SP6 was inhibited while the amino acid metabolism in TS-SP6 was activated. Like the TCA cycle, amino acid metabolism showed different changes in different growth and development stages of the same variety, and different changes in different varieties in the same growth and development stage. It was worth noting that amino acid metabolism and TCA cycle showed opposite activation or inhibition patterns in different rice at the same period, which indicated that there may be a certain balance between amino acid metabolism and TCA cycle. The intermediate metabolites in TCA cycle can be converted to some amino acids, resulting in the decrease of intermediate metabolites in TCA cycle. When the intermediate metabolites in TCA cycle decrease, amino acids can be converted into key products of TCA cycle under the catalysis of enzymes to ensure the rate of TCA cycle. This reasonably explained the main reason for the opposite change between amino acid metabolism and TCA cycle in this study.

Glutamic acid, proline and arginine can be converted into alpha-ketoglutarate (Figure 14), which affected the reaction activity of TCA. It also explained that the content of alpha-ketoglutarate increased in TLP-SP3 when the intermediate metabolites of TCA cycle were reduced (Figure 13A). Proline has been proved to respond to abiotic stresses. The catabolism of proline in mitochondria can transfer electrons to respiratory chain to promote the production of ROS and ATP, and this process was intensified under drought stress (Liang et al., 2013; Schertl and Braun, 2014). But when plants grew in adversity for a long time, they can quickly remove excess proline by increasing the amount of catabolic enzymes, and prevent invalid circulation by post translational regulation (Batista-Silva et al., 2019). This explained the difference of proline content between the two rice varieties in different growth and development stages, as well as the difference of ROS in previous work. On the other hand, it also showed that there were differences in the mechanism of response to spaceflight between the two rice varieties.

Glutamate decarboxylase catalyzed the decarboxylation of glutamate to produce GABA. GABA in plants can respond to biotic and abiotic stresses such as temperature, dehydration, salinity, oxygen stress, mechanical damage, acidosis, and virus infection (Bouché and Fromm, 2004). GABA was also a signaling molecule. It can participate in the control of C:N balance in plants through direct interaction with glutamate receptors (signaling) or by controlling the utilization of glutamate (metabolic) (Kang and Turano, 2003). Recent studies have shown that GABA can affect the development of plant roots and can negatively regulate the content of malic acid in plants (Ramesh et al., 2015). In our study, GABA content changed significantly in TLP-SP6 and TS-SP6. Therefore, this may also be one of the reasons for the changes in their malic acid content, which also showed that GABA can affect the activity of TCA by adjusting the malic acid content.

### The Effect of Spaceflight on Other Metabolic Reactions of Two Rice Varieties

Soluble sugars especially sucrose, glucose and fructose play a central role in maintaining plant cell structure and metabolism. They are involved in the response to various abiotic stresses, acting as signal molecules for nutrients and metabolites and activating or interacting with specific plant hormone transmission pathways, resulting in changes in gene expression and protein abundance (Couée et al., 2006). The changes of these sugars were also found in our metabolome results (Supplementary Tables 2–5). Compared with the TS-CK3, fructose and glucose increased by 1.63 times and 1.91 times (Supplementary Table 3), respectively in the TS-SP3 while the sucrose content did not change, but the sucrose content of these three sugars have only reduced by 83% in TLP-SP3 (Supplementary Table 2). The content of these three sugars in the TS-SP6 was significantly lower than that of the TS-CK6, while only the sucrose content in TLP-SP6 increased by 1.5 times, and the concentration of the other two sugars decreased (Supplementary Table 4). Previous studies have found that changes in sugar concentration showed unique expression patterns under different abiotic stresses and in different species. For example, drought stress, salt stress, and low temperature stress would increase sugar concentration, while heavy metals, malnutrition, and ozone would reduce sugar concentration (Prado et al., 2000; Gill et al., 2003; Rosa et al., 2004, 2009). These results proved that the spaceflight caused the response of rice to stress, and the response pattern of the two types of rice was different. Other studies have shown that the sugar accumulation caused by the sugar disturbance caused by the stress response would also limit the growth of rice seedlings (Mishra and Dubey, 2013). This may also be the reason for the inconsistent plant weights of the two rice plants in our study. Our research also found some other carbohydrate changes (Supplementary Tables 2–5). Mannose and raffinose can protect plant cells from oxidative damage caused by various stress conditions, which has been determined that they have the ability to eliminate ROS (Nishizawa et al., 2008). The mannose was accumulated in TLP-SP3, TLP-SP6, TS-SP3, and TS-SP6, while the concentration of raffinose decreased (Supplementary Tables 2–5). It was reported that drought stress (Shahbazy et al., 2020) and salt stress (Kumari and Parida, 2018) have disturbed the changes of D-tagatose. Our results showed that the D-tagatose was decreased in the TLP-SP3 and TS-SP3, and was accumulated in the TS-SP6. Moreover, the concentrations of glucose, sucrose, maltotriose, rhamnose, and raffinose had different trends in the two rice varieties after spaceflight. These evidences suggest that there was a differential metabolic rearrangement of carbohydrate metabolites in different varieties and different growth stages. In addition, our research also provided the change pattern of carbohydrate metabolites in rice after spaceflight for the first time.

Many studies have shown that vitamins can reduce the damage caused by abiotic stress to plants (Taffouo et al., 2009; Asensi-Fabado and Munné-Bosch, 2010). Abiotic stress led to a lack of vitamins and cofactors, which would reduce the growth performance of plants, and supplementing plants with the lack of vitamins under stress conditions would improve their growth performance (Hanson et al., 2016). Niacinamide is the common form of vitamin B3 (niacin), which is the active part of the coenzymes NAD(P) and NAD(P)H. It participated in the redox reaction of various enzymes and played an important role in the energy metabolism of the organism (Hunt et al., 2004). Studies have proved that nicotinamide can respond to the oxidative stress response of UV-B to *Pisum sativum* (Berglund et al., 1996), and metabolize nicotinamide into hydrochloric acid when in plants. Furthermore, Niacinamide and hydrochloric acid can prevent DNA breakage and cell electrolyte leakage caused by oxidative stress, and induced the accumulation of glutathione, aconitase and fume at the same time (Berglund et al., 2017). Compared with the control group, nicotinamide decreased by 55 and 54% in TLP-SP3 and TS-SP6, respectively, but decreased by 33% in TLP-SP6, and increased by 1.57 times in TS-SP3 after spaceflight (Supplementary Tables 2–5). In addition, it was found that niacin had different trends in the TLP of SP3 and SP6 (Supplementary Tables 2, 4), which may be caused by the different activities of nicotinamides in the two kinds of rice. This may also explain the changes in aconitase and fume content in our research. Pyridoxal and pyridoxine were two forms of VB6 (Czégény et al., 2019). Pyridoxine can regulate the tolerance of plants to abiotic stress and affect root development (Chen and Xiong, 2005). Our study showed that pyridoxine accumulated 3.08 times and 3.95 times in TLP-SP3 and TS-SP6 (Supplementary Tables 2, 5), respectively. Moreover, we also found that pyridoxal accumulated 1.34 times and 1.30 times in TLP-SP3 and TLP-SP6 (Supplementary Tables 2, 4), respectively. Our results showed that the content of pyridoxine increased when SP3 rice developed from TLP to TS, but it showed the opposite trend in SP6. These kinds of vitamins showed different trends in the two kinds of rice, which indicated that the vitamin metabolism of the two kinds of rice showed different expression patterns to the spaceflight. What’s more exciting was that these vitamins can respond to the oxidative stress effect of abiotic stress. Therefore, our results implied that the spaceflight had an oxidative stress effect on the two kinds of rice, and this effect continued until the TS period of rice.

## Conclusion

This study investigated the metabolic disturbances in different growth and development stages of rice during the planting process of rice seeds returned to the ground after spaceflight, and compared the differences in the metabolic level of the two types of rice in response to the spaceflight. Our research showed that spaceflight causes the stress response of rice seeds and leads to different changes in their metabolic characteristics at different growth and development stages during ground planting. DN3 and DN6 adapted to changes in the spaceflight by remodeling metabolic pathways, but their mechanisms had certain differences. The intermediate metabolites of TCA cycle and amino acid metabolism in DN3 and DN6 have changed significantly. We further confirmed the effect of spaceflight on TCA cycle and amino acid metabolism by using qRT-PCR and metabolomics results. In DN3, the TCA circulation rate decreases while the amino acid metabolism rate increases during TLP. The TCA cycle rate of DN3 increased in TS while the amino acid metabolism rate did not change. In DN6, we observed the increase of TCA rate and the decrease of amino acid metabolism rate in TLP. However, the TCA was inhibited and the amino acid metabolism rate was increased in TS-DN6. These results provided useful insights into the molecular mechanism of different biological effects of spaceflight on rice seeds. Our research focuses on the changes in rice metabolites at different developmental stages after rice seeds return to the ground through spaceflight, which provides new insights for space biology requirements in exploration.

## Data Availability Statement

The original contributions presented in the study are included in the article/Supplementary Material, further inquiries can be directed to the corresponding author/s.

## Author Contributions

WL and JC designed of the study. DZ conducted the experiments and analyzed the data. DZ and YY wrote the manuscript. YX and ML revised the manuscript. SG conducted field planting of experimental materials. DC provided the experimental site. YS guided the experiments. All authors read and approved the manuscript in its final form.

## Conflict of Interest

The authors declare that the research was conducted in the absence of any commercial or financial relationships that could be construed as a potential conflict of interest.

## Footnotes

1 http://www.metaboanalyst.ca/

## Abbreviations

TLP: Three-Leaf Stage
TS: Tillering Stage
SJ-10: ShiJian-10 retractable satellite
TCA: Tricarboxylic acid cycle
DN6: Dongnong416
DN3: Dongnong423
SP: Spaceflight group
CK: Control group
TLP-SP3: DN3 rice spaceflight treatment group at three-leaf stage
TS-CK3: DN3 rice control group at three-leaf stage
TLP-SP6: DN6 rice space flighttreatment group at three-leaf stage
TS-CK6: DN6 rice control group at three-leaf stage
EL: Electrolyte leakage rate
POD: Peroxidase
CAT: Catalase
SSC: Soluble sugar content
PLS-DA: Partial least squares discriminant analysis
PCA: Principal component analysis
RT-qPCR: Real-time quantitative PCR.
