# Supplementary material for: Metabolomics Analysis in Different Development Stages on SP0 Generation of Rice Seeds After Spaceflight
Source: Front Plant Sci. 2021 Jun 30;12:700267. doi: 10.3389/fpls.2021.700267 (PMC8278407; doi:10.3389/fpls.2021.700267)
Supplement: Supplementary file 1 [file Table_1.DOCX]

**Table S1. Primers used in the qRT-PCR analysis.**

| ID | Forward primer (5’–3’) | Reverse primer (5’–3’) |
| --- | --- | --- |
| LOC107280261 | GGGCGGGCTGATGAACAAGATG | CAGCGTGGAGCGTATGATGGC |
| LOC4333381 | CAGGGAGCAGGGAGTGGAGAC | TTGTCGTCCTTGGGAATTGGTTGG |
| LOC4336581 | GCATAGCGGCTGGCACATCG | ACCATCACCAGTTGCTCCACATG |
| LOC4332506 | CCTTCGGCGTCCTCCTCTCC | CTTCTCGGCGGCTTCAGTCTTG |
| LOC4340706 | CCTTGATCCATCTGGTCGTGCTG | ACAGGATTGGCAGAGGCAGGAG |
| LOC4352605 | GCAGCCGATGGTGGGTCAATC | AGCATGGCACTCACAGGAACAAC |
| LOC4325651 | TCCCCGCCATCGTGTTCCTC | AGTCCGTGTCCAGCGAGAGC |
| LOC4339521 | GTCAAGCACTGCGGCATCTCC | AGGCGGTTCACCTGGCTGAC |
| LOC4325621 | CGCCGACTCCTCCCTCCAAG | CGACGCCGAGATTGACCTTCAC |
| LOC4328828 | TGCCAGCGGTGATCCAGAGAG | CCCTTTGCCCATACAGTCCCATG |
| LOC4331017 | CCTTCGCCGTCTCCTCCTCAC | CAGTCCTCGCCTCGCTCCTC |
| LOC4332108 | CGACTGCTACACGCCACAAGG | TGGCACCTCAGCAACAACATCAG |
| LOC4330649 | CCGCCGACATCAACACCTTCAG | TGCCGTTCTGCTCCGTCTCC |
| LOC4333896 | GCAATGGCGGCTACGAGGTG | AAGTTGTTGATGTCGGCGGTCTC |
| LOC4337272 | CATCGCCGAGTACATCTGGGTTG | GCCCTGTGCTTGATCCATCGTAG |
| LOC4326958 | CTGAGCGTGGTGATGAAGTTCGG | CGAGGAGGAGGAGATTGGTGGTC |
| LOC4334188 | GCCGTCGTCGTGGACAACAG | AGGCAGATGATGGTGGAACAGTTC |
| LOC4348992 | CACCGTCATGCTCCCGAGATTG | AGTCACTCCAGGTGCCATTTCAAC |
| LOC4335317 | TGGCTCAGCTTGGAGTGGTAAGG | GTGCCTCGTTCTCACCCACAAAG |
| LOC4346272 | CACTGTGGTCAATGCCGATCTCAG | TCTACCGTCCGAATGGGCTCTTC |
| LOC4326980 | GAAGGTGGTGGCTGAGAAGGAAAC | GTGGGATGAGCGTGGCATGTAC |
| LOC4326849 | CGAAGGCTCTGGCTGAGAATGAAG | TTCCGAGGGCAATGGCGTTTG |
| LOC4337862 | CACAGGACGGGCACCATTCAC | ACTCGCATTCGCTCATCAACAGG |
| LOC4329234 | ACACTTGCTGCTTCGTCAATAGGG | TCTGCCATGCTGCCATCAACTAC |
| LOC4349660 | GGTCTCCAACTTCCGTCGCATAAG | ACCACTCAGACTGCCAACAATACG |
| LOC4351381 | CCTCCTCCGCCACATCCTCTC | GTCGTCGAAGCCGTTGGAGTG |
| LOC4324260 | GGATGTTGGTGGTAGCCGATCAAG | CCTTCCTCGTGCTGCTCAATCTG |
| LOC4327968 | GGAGGCAAGTGGAGGTTGAGTATG | TTCAGCCGTGTAATCCCAGCATTC |
| LOC4336402 | CCTCATCTCCGTCCTCGTCCAC | TCCCGCCGTTCTCGTAGATATGG |
| LOC4338868 | ACGGATGCGGCTTGTTGCTG | CCTTCGCTGCCACAAGACCAC |
| LOC4350456 | CATTGGCTCCTGGCGGTGTC | GCTTGCCGTGCTCCTTCTGG |
| LOC4352058 | TACCACCACCACCCACAAGAGTC | CACTGCGAAGTTGATCCTGTCCTC |
| LOC4334425 | CGCTGTTGAGGAATGAGGCTGTC | CATCCTCTCTGTTGGTGTGGTTGG |
| LOC4333898 | AATTGCTGGGATCGCTGCCTATG | TGACCATGACACAAGGACAATGCC |
| LOC4329938 | GCGACGAGAGCGGGATGATAAAC | CGGAGACGACAATGGTGAACATGG |
| LOC4329450 | GAAGTCCGTGCCGCCATCAAG | GCCATCACCATCCAGGATCATGTC |
| LOC4339677 | AAGCATTCGTGACAGTGGACAAGG | GTTGGCAGGCACGGAGATGTC |
| LOC4346318 | GCAGGTGCGTGTGGTGGTATG | CAGCAAGACGGCATTCCTCTAGC |
| LOC4339583 | TGCTGTCGTAGTGTCTCCTGTGG | CAATCTTCTGCCTGGCCCATCTG |
| LOC4331322 | TCTCTCCACGCCGTCGCTTC | GTCCAAATCAGCCACCGCAGAG |
| LOC4326891 | CTCCAACTTCGTCGTCGTCCATG | AGCCACAAAGCCAGCCTTCAC |
| LOC4333554 | CCCTGCTTCTGCTTACGACATCTG | AGCACATCGCTGAACGCCTTC |
| LOC4334912 | TTCTTCACCTTGACGCACATCCAG | AGCCTCCTAGCATAACCTCCTTCC |
| LOC4325755 | CTGTGAAGCCGCAGATCGTAAGG | AGCAGCAATGGACACCAGAAGC |
| LOC4351460 | CACTCCTTCAACCGGGCATTCAG | CTTGTTCAGGGCACTCAGGTAAGC |
| LOC4328147 | TGCCAAGCCACATTCACCTGAC | TTCGCAGCAGCAAAGAAGTCCTC |
| LOC4330192 | TTGGAGCTAGCGGACACTGG | CAGCTTGTACTGCTTGCCGG |
| LOC4335977 | CAAGCCCAACATCGTGACGG | GGTCCATCACGTAGCAGCCT |
| LOC4333564 | GTGCTTGTCGTCGCAGGATG | ATTTGCTCAACGGCAGCCAC |
| LOC4340640 | GCGACTATGTGCCAGGATTGAAGG | TGTCCGTGGTATCTGTAGGTGTCC |
| LOC4352803 | CCTCCGCCAAGCCCAGATCC | GCATCCGCACTCGCTGTAACC |
| LOC4330673 | CGTTTGCCCTCTATGGTGATGGTG | TGCCCTCCACTCAGCGGTTC |
| LOC4346159 | GAGAGGATTGCTGGAGCTGATGTG | GCTGCCATTGGTACTGCTCTGTAG |
| LOC4334968 | GCCGTGGACAAGGTGGTTCTATG | CAGCACCAGTAGCAACAGGGATG |
| LOC4347022 | GCCTTCTTACGCCTGTCTTGGAG | ATTCGTCTGGTTGGAGCTGCTTC |
| LOC4343003 | CGCCAATACCACAACGTGAACATC | CACCTCCTCCGCAATCATACCAAG |
| LOC4327289 | GGCATCGCACAACAGGCTGAG | CAGGCAAGTTGAGAAGCGGAGAG |
| LOC4350660 | TATGGTCCTTGGCGGGATGAGAG | TTGGCAGCACTTTCTGGCACTC |
| LOC4328812 | CCTCGCATGGCTGGTTGGTTAG | TGGGACTCGTTCTCTGACAGGTG |
| LOC9272485 | CTCCGCCCAGGTTACACAATCG | GTCCGCTCTTGCCACGCTTC |
| LOC9272295 | CTCCGCCCAGGTTACACAATCG | GTCCGCTCTTGCCACGCTTC |
| LOC9270306 | TGTTCTTGCTGGTGCTGAGTATGG | CAGGTTGCTTCGGTGGATACGC |
| LOC4331547 | TGGCTGGTGCTGAGTACGGTAG | AGGTTGCTGCGGTGAATCCTTTC |
| LOC4329858 | CGTTGCTCTGCTCCTCTCATCTG | CTTCCTGCGTGGTGCTGCTG |
| LOC4324442 | GCTGTTCACGGTTCTGCTCCTG | CTCGGAGATGGTCTGGAGGATGG |
| LOC4324176 | GTTGGAACCGTGGAATCTGGGAAG | CGCAGCAACCGCATCAATGAAC |
| LOC4344403 | GCCTTGTCGTTCTACTGCCTCATG | ATCTGCTTGCGGAGTGTTGGTTC |
| LOC4335689 | GGTCTTGTGGTGCCTGTTATCCG | GGTGAATGTTCCTCCTGCCATCTC |
| LOC4335673 | CACCAGCAGCGTGTACCTTGAG | CCCAGATATGCCAGGCGAAGTTG |
| LOC4330016 | AAGGCACCAATGTTGACCAAGGG | CTCAGCCGCATCATCAAGATCCTC |
| LOC4343710 | ATGGTGAAAGTGAAGGCTGCTCTG | CGACCTGGCTTGTGGATGTAACC |
| LOC4344541 | CCAAGCTCGACGGCATGTACG | TGGTCACGGCTGTCCTGTATCC |
| LOC4345159 | GGCCTCCCGTTCAAAGTTATCTGG | TCCTCTGGAGACTTGAGCCTTGC |
| LOC4346890 | TCGACCTGACCAGCTTCTACCAG | CGTCCAGCCTCTTCCTCTCCTC |
| LOC4343404 | GCAAACCGCCCACTGTCACC | AGGCTGAATTTGGTGGCAAGAGG |
| LOC4342350 | CACTGGAGATGGCAACGCTATGG | TACCACCTTCACCACGGGAACC |
| LOC4328125 | CTCTGTCGGCTCCTGTTGCTAAC | GAACTTGGTGGCGAGAGGAATGG |
| LOC4350629 | TCACCACCACCACCCAGCAG | GTCCCGAGCACCAGGTCCAG |
| LOC4344494 | GCACTGGGCGAAGAACTTCTCC | CGTCGGCATCGGTCCACTTG |
| LOC4335816 | CGGCGATATCTGACCCAGAAGTTG | GCCTCTTTCCCAGCCTTATCATCC |
| LOC112939253 | TCGCTGCTCTCCTCCACCATC | ATCGCCGCTCTGTCCTGCTG |
| LOC112939252 | TCTCCTCCACCGTCGTCATCTG | GCTGCTCTGTCCTGTTGCCTAAG |
| LOC4346939 | CGCTTTTGGAGGAGGATACTGCTC | TGCCAGTGCAACGATCTCATAGG |
| LOC4332774 | TTGGACATAAGCGTGGCGAGAAG | GATCTCTGTGGCTGCTGCTATGTG |
| LOC4326249 | CACCATCCTGCCACTGTTCTCAC | CAGAGCCCTTTCCAGCCTTTGC |
| LOC4336595 | CAACCACTCCTCCACGCAGTTC | AACTCCTCCCTCAGCCATATCTCG |
| LOC4339682 | GCAGCGGAGGTGTTCAAGAAGG | GCCACCAACCACAGGGACATTC |
