# Supplementary material for: Metabolomics Analysis in Different Development Stages on SP0 Generation of Rice Seeds After Spaceflight
Source: Front Plant Sci. 2021 Jun 30;12:700267. doi: 10.3389/fpls.2021.700267 (PMC8278407; doi:10.3389/fpls.2021.700267)
Supplement: Supplementary file 6 [file Table_6.docx]

Table S6 Changes in gene associated with TCA and amino acid metabolic in the three-leaf stage (TLP) and the tillering stage (TS) as identified by qRT-PCR.

| ID | Describe | DN3 | | | | | | DN6 | | | | | |
| --- | --- | --- | --- | --- | --- | --- | --- | --- | --- | --- | --- | --- | --- |
|  |  | TLP | TLP | TLP | TS | TS | TS | TLP | TLP | TLP | TS | TS | TS |
| LOC107280261 | L-asparaginase 2 | 8.24 | 11.46 | 10.07 | 1.21 | 1.03 | 0.94 | 0.45 | 0.24 | 0.33 | 4.02 | 5.13 | 2.93 |
| LOC4333381 | L-asparaginase 1 | 3.99 | 2.91 | 2.53 | 2.02 | 2.37 | 2.95 | 0.17 | 0.21 | 0.31 | 4.8 | 5.25 | 6.07 |
| LOC4336581 | L-asparaginase 3 | 0.79 | 0.62 | 0.49 | 1.32 | 1.5 | 1.1 | 0.96 | 0.8 | 1.09 | 0.22 | 0.4 | 0.21 |
| LOC4332506 | asparagine synthetase 1 | 2.14 | 2.32 | 2.1 | 0.29 | 0.22 | 0.17 | 2.01 | 1.22 | 1.39 | 3.36 | 4.42 | 6.25 |
| LOC4340706 | asparagine synthetase 2 | 9.22 | 12.92 | 8.97 | 0.74 | 0.47 | 0.39 | 0.09 | 0.22 | 0.16 | 4.2 | 2.36 | 2.6 |
| LOC4352605 | asparagine synthetase domain-containing protein 1 | 5.15 | 5.08 | 5.01 | 1.03 | 0.8 | 1.28 | 1.73 | 1.42 | 1.98 | 0.49 | 0.62 | 0.59 |
| LOC4325651 | Tyrosine aminotransferase | 4.43 | 2.82 | 4.94 | 0.31 | 0.56 | 0.25 | 0.13 | 0.13 | 0.16 | 0.71 | 0.87 | 0.97 |
| LOC4339521 | Tyrosine aminotransferase | 1.23 | 2.2 | 1.76 | 0.3 | 0.25 | 0.36 | 1.82 | 2.06 | 1.99 | 0.18 | 0.22 | 0.25 |
| LOC4325621 | aspartate aminotransferase | 7.98 | 10.81 | 6.1 | 0.73 | 0.41 | 0.33 | 0.05 | 0.08 | 0.09 | 8.66 | 7.71 | 11.92 |
| LOC4328828 | aspartate aminotransferase | 3.42 | 3.2 | 3.06 | 15.57 | 7.1 | 10.81 | 0.74 | 0.41 | 0.36 | 11.74 | 8.19 | 7.01 |
| LOC4331017 | aspartate aminotransferase | 2.03 | 3.02 | 2.2 | 5.83 | 3.5 | 6.71 | 0.54 | 0.97 | 0.71 | 0.8 | 0.39 | 1.03 |
| LOC4332108 | glutamine synthetase | 0.38 | 0.32 | 0.48 | 0.44 | 0.26 | 0.25 | 0.34 | 0.37 | 0.42 | 4.05 | 2.21 | 1.75 |
| LOC4330649 | glutamine synthetase | 4.88 | 2.81 | 5.31 | 0.72 | 0.97 | 0.78 | 0.51 | 0.67 | 0.64 | 2.03 | 2.72 | 1.98 |
| LOC4333896 | glutamine synthetase | 8.8 | 9.67 | 5.14 | 0.12 | 0.19 | 0.11 | 0.35 | 0.2 | 0.42 | 3.47 | 2.29 | 3.2 |
| LOC4337272 | Glutamate synthetase | 0.48 | 0.32 | 0.45 | 1.15 | 1.16 | 0.97 | 1.09 | 0.45 | 0.83 | 0.39 | 0.37 | 0.4 |
| LOC4326958 | aspartokinase 2 | 2.81 | 2.4 | 3.36 | 0.11 | 0.02 | 0.14 | 0.33 | 0.2 | 0.1 | 8.43 | 8.17 | 17.54 |
| LOC4334188 | Aspartate semialdehyde dehydrogenase | 1.75 | 2.15 | 1.53 | 1.78 | 2.35 | 2.62 | 0.16 | 0.13 | 0.11 | 21.48 | 15.89 | 14.14 |
| LOC4348992 | Aspartate semialdehyde dehydrogenase | 3.26 | 5.08 | 4.59 | 0.98 | 0.85 | 0.66 | 0.64 | 1.19 | 0.68 | 0.16 | 0.2 | 0.15 |
| LOC4335317 | Dihydropicolinate synthase | 2.33 | 1.56 | 2.05 | 0.83 | 0.67 | 0.54 | 65.09 | 82.96 | 69.76 | 0 | 0.01 | 0 |
| LOC4346272 | Dihydropicolinate reductase | 0.6 | 0.5 | 0.34 | 0.95 | 0.5 | 0.31 | 0.49 | 0.72 | 0.6 | 0.13 | 0.18 | 0.16 |
| LOC4326980 | Diaminopimelate dehydrogenase | 8.05 | 9.54 | 10.68 | 0.1 | 0.06 | 0.09 | 1.34 | 0.99 | 1.54 | 3.83 | 4.88 | 4.36 |
| LOC4326849 | Diaminopimelate dehydrogenase | 2.83 | 1.79 | 2.49 | 0.58 | 0.55 | 0.85 | 0.24 | 0.66 | 0.31 | 7.9 | 6.68 | 5.73 |
| LOC4337862 | Diaminopimelate dehydrogenase | 1.25 | 1.55 | 0.99 | 0.22 | 0.52 | 0.31 | 3.31 | 2.03 | 2.63 | 4 | 3.27 | 2.53 |
| LOC4329234 | Diaminopimelate decarboxylase | 1.16 | 2.63 | 1.63 | 0.11 | 0.25 | 0.13 | 1.21 | 1.06 | 0.83 | 0.07 | 0.08 | 0.07 |
| LOC4349660 | Homoserine dehydrogenase | 3.53 | 4.07 | 6.26 | 0.25 | 0.13 | 0.14 | 0.12 | 0.12 | 0.14 | 4.14 | 1.57 | 3.02 |
| LOC4351381 | Homoserine dehydrogenase | 2.02 | 1.54 | 1.72 | 1.37 | 1.2 | 0.99 | 0.23 | 0.25 | 0.22 | 2.12 | 1.07 | 1.06 |
| LOC4324260 | Homoserine kinase | 9.55 | 3.96 | 7.69 | 0.13 | 0.09 | 0.17 | 0.13 | 0.25 | 0.11 | 4.12 | 2.42 | 7.13 |
| LOC4327968 | Homoserine kinase | 0.43 | 0.4 | 0.4 | 0.56 | 0.41 | 0.42 | 1.2 | 0.87 | 0.76 | 8.77 | 8.89 | 14.44 |
| LOC4336402 | probable low-specificity L-threonine aldolase 1 | 1.21 | 1.09 | 0.92 | 1.55 | 0.95 | 0.92 | 0.17 | 0.5 | 0.29 | 0.04 | 0.04 | 0.05 |
| LOC4338868 | Glycine hydroxy-methyl transferase | 8.97 | 3.64 | 10.47 | 0.31 | 0.2 | 0.36 | 0.04 | 0.02 | 0.02 | 37.04 | 36.25 | 4.96 |
| LOC4350456 | Glycine hydroxy-methyl transferase | 2.1 | 1.57 | 2.04 | 0.18 | 0.2 | 0.06 | 13.31 | 11.57 | 6.7 | 0.78 | 1.32 | 0.8 |
| LOC4352058 | Glycine hydroxy-methyl transferase | 0.11 | 0.13 | 0.08 | 3.95 | 3.2 | 4.13 | 0.21 | 0.29 | 0.42 | 1.09 | 0.7 | 1.51 |
| LOC4334425 | tryptophan synthase alpha chain | 0.26 | 0.15 | 0.3 | 0.4 | 0.18 | 0.28 | 1.42 | 0.57 | 0.6 | 2.09 | 3.81 | 2.28 |
| LOC4333898 | Tryptophan synthase | 0.45 | 0.35 | 0.41 | 0.11 | 0.14 | 0.06 | 0.58 | 0.32 | 0.38 | 0.1 | 0.1 | 0.16 |
| LOC4329938 | Acetolactate synthase | 4.02 | 7.06 | 5.21 | 0.42 | 0.32 | 0.25 | 0.39 | 0.51 | 0.58 | 3.31 | 2.41 | 2.04 |
| LOC4329450 | Acetolactate synthase | 3.11 | 4.94 | 3.61 | 0.11 | 0.2 | 0.17 | 1.86 | 1.77 | 1.14 | 2.59 | 2.43 | 1.93 |
| LOC4339677 | Ketol-acid reductoisomerase | 0.2 | 0.29 | 0.18 | 0.59 | 0.49 | 0.37 | 1.27 | 1 | 1.01 | 0.13 | 0.08 | 0.08 |
| LOC4346318 | Phosphogluconate dehydratase | 1.64 | 2.11 | 2.39 | 0.41 | 0.47 | 1.06 | 2.77 | 2.75 | 3.16 | 3.28 | 4.07 | 5.84 |
| LOC4339583 | branched-chain-amino-acid aminotransferase 5 | 3.67 | 7.12 | 2.42 | 4.76 | 4.29 | 9.02 | 0.27 | 0.18 | 0.12 | 1.81 | 2.78 | 0.82 |
| LOC4331322 | branched-chain amino acid aminotransferase 2 | 17.3 | 20.57 | 26.39 | 0.14 | 0.11 | 0.08 | 1.27 | 1.43 | 0.95 | 2.02 | 1.69 | 3.37 |
| LOC4326891 | Glutamate 5-kinase | 2.81 | 3.56 | 2.87 | 0.45 | 0.19 | 0.15 | 0.39 | 0.2 | 0.49 | 0.01 | 0.02 | 0.01 |
| LOC4333554 | Ornithine--oxo-acid transaminase | 0.5 | 0.25 | 0.43 | 0.62 | 1.05 | 1.67 | 6.59 | 9.2 | 5.47 | 0.06 | 0.1 | 0.15 |
| LOC4334912 | arginase 1, mitochondrial-like | 1.34 | 1.87 | 1.57 | 0.04 | 0.08 | 0.04 | 0.13 | 0.23 | 0.15 | 2.67 | 2.7 | 3.86 |
| LOC4325755 | pyrroline-5-carboxylate reductase | 0.11 | 0.24 | 0.1 | 1.2 | 1.37 | 1.85 | 5.91 | 6.43 | 7.03 | 0.89 | 0.93 | 0.99 |
| LOC4351460 | 2-isopropylmalate synthase A | 0.42 | 0.35 | 0.43 | 0.37 | 0.43 | 0.47 | 0.69 | 0.3 | 0.32 | 0.29 | 0.32 | 0.33 |
| LOC4328147 | 3-isopropylmalate dehydratase large subunit, chloroplastic | 0.66 | 1.24 | 1.26 | 0.42 | 0.49 | 0.25 | 0.08 | 0.1 | 0.09 | 7.25 | 7.49 | 10.93 |
| LOC4330192 | 3-isopropylmalate dehydratase small subunit 3 | 1.6 | 2.11 | 1.15 | 1.46 | 1.21 | 1.06 | 0.69 | 0.76 | 0.35 | 0.64 | 0.4 | 0.48 |
| LOC4335977 | glutamate decarboxylase 4 | 8.57 | 7.92 | 12.59 | 0.53 | 0.53 | 1.17 | 7.69 | 6.92 | 4.87 | 15.06 | 14.23 | 13.34 |
| LOC4333564 | pyruvate dehydrogenase E1 component subunit beta-4 | 1.01 | 1.11 | 1.84 | 10.64 | 8.01 | 12.17 | 0.08 | 0.18 | 0.09 | 1.05 | 1.31 | 1.79 |
| LOC4340640 | pyruvate dehydrogenase E1 component subunit alpha-2 | 0.52 | 0.12 | 0.31 | 4.85 | 3.1 | 3.95 | 0.21 | 0.33 | 0.2 | 0.42 | 0.46 | 0.34 |
| LOC4352803 | pyruvate dehydrogenase E1 component subunit beta-3 | 0.56 | 1.52 | 1.3 | 2.11 | 2.69 | 3.58 | 3.15 | 2.86 | 3.68 | 0.26 | 0.14 | 0.28 |
| LOC4330673 | pyruvate dehydrogenase E1 component subunit alpha-1 | 0.18 | 0.05 | 0.09 | 1.24 | 1.27 | 1.67 | 1.7 | 1.93 | 2.34 | 0.16 | 0.07 | 0.18 |
| LOC4346159 | pyruvate dehydrogenase E1 component subunit beta-1 | 0.35 | 0.33 | 0.22 | 0.77 | 1 | 0.91 | 5.29 | 3.64 | 5.04 | 0.04 | 0.04 | 0.01 |
| LOC4334968 | pyruvate dehydrogenase E1 component subunit alpha-3 | 0.05 | 0.03 | 0.04 | 1.13 | 1.47 | 0.96 | 0.42 | 0.36 | 0.14 | 1.03 | 0.78 | 1.31 |
| LOC4347022 | dihydrolipoyllysine-residue acetyltransferase component 4 | 0.56 | 1.05 | 1.26 | 0.79 | 0.86 | 1.09 | 3.52 | 5.5 | 3.73 | 0.17 | 0.14 | 0.14 |
| LOC4343003 | dihydrolipoyllysine-residue acetyltransferase component 3 | 0.96 | 1.11 | 1.26 | 0.74 | 0.54 | 0.81 | 3.79 | 3.08 | 3.42 | 0.11 | 0.14 | 0.1 |
| LOC4327289 | lipoamide acyltransferase component of branched-chain alpha-keto acid | 1.1 | 1.01 | 1.18 | 0.19 | 0.28 | 0.29 | 0.2 | 0.11 | 0.15 | 0.22 | 0.27 | 0.07 |
| LOC4350660 | citrate synthase 4 | 7.56 | 8.21 | 8.62 | 1.84 | 2.5 | 2.33 | 1.27 | 2.24 | 1.5 | 0.36 | 0.3 | 0.35 |
| LOC4328812 | citrate synthase 3 | 0.36 | 0.74 | 0.53 | 3.23 | 11.73 | 4.77 | 6.12 | 6.64 | 3.68 | 0.08 | 0.1 | 0.17 |
| LOC9272485 | ATP-citrate synthase subunit alpha chain protein 1 | 0.32 | 0.35 | 0.34 | 3.12 | 2.88 | 2.23 | 16.26 | 19.37 | 11.17 | 0.33 | 0.39 | 0.66 |
| LOC9272295 | ATP-citrate synthase alpha chain protein 2 | 0.58 | 0.53 | 0.82 | 0.34 | 0.19 | 0.25 | 11.86 | 14.9 | 12.53 | 0.09 | 0.06 | 0.11 |
| LOC9270306 | putative aconitate hydratase | 0.58 | 0.83 | 0.66 | 2.01 | 3.86 | 2.19 | 3.28 | 2.53 | 4.21 | 0.11 | 0.12 | 0.18 |
| LOC4331547 | putative aconitate hydratase | 1.03 | 1 | 1.64 | 4.6 | 3.72 | 5.76 | 4.41 | 6.04 | 3.72 | 0.19 | 0.3 | 0.4 |
| LOC4329858 | isocitrate dehydrogenase [NAD] regulatory subunit 1 | 3.38 | 3.19 | 2.39 | 3.37 | 5.86 | 5.14 | 2.06 | 2.87 | 3.44 | 0.28 | 0.36 | 0.47 |
| LOC4324442 | isocitrate dehydrogenase [NAD] catalytic subunit 5 | 1.78 | 1.53 | 2.23 | 0.11 | 0.13 | 0.24 | 1.47 | 1.03 | 1.23 | 0.25 | 0.18 | 0.16 |
| LOC4324176 | cytosolic isocitrate dehydrogenase [NADP] | 0.41 | 0.65 | 0.55 | 1.53 | 0.48 | 0.52 | 0.1 | 0.18 | 0.12 | 0.29 | 0.26 | 0.23 |
| LOC4344403 | 2-oxoglutarate dehydrogenase | 0.31 | 0.45 | 0.52 | 8.4 | 5.27 | 12 | 4.92 | 4.48 | 3.47 | 0.94 | 0.55 | 1.06 |
| LOC4335689 | dihydrolipoyllysine-residue succinyltransferase component of 2-oxoglutarate dehydrogenase complex 1 | 0.28 | 0.33 | 0.3 | 0.96 | 0.68 | 0.54 | 0.89 | 0.43 | 0.62 | 0.5 | 0.6 | 0.47 |
| LOC4335673 | 2-oxoglutarate dehydrogenasel | 1.54 | 1.7 | 1.62 | 0.25 | 0.66 | 0.4 | 0.42 | 0.25 | 0.33 | 0.28 | 0.17 | 0.22 |
| LOC4330016 | succinate--CoA ligase [ADP-forming] subunit beta | 0.18 | 0.19 | 0.3 | 2.37 | 2.13 | 2.65 | 8.88 | 10.1 | 7.4 | 0.08 | 0.08 | 0.09 |
| LOC4343710 | succinate--CoA ligase [ADP-forming] subunit alpha | 0.3 | 0.18 | 0.47 | 1.78 | 1.59 | 1.34 | 7.37 | 5.51 | 5.05 | 0.11 | 0.11 | 0.28 |
| LOC4344541 | succinate dehydrogenase [ubiquinone] iron-sulfur subunit 1 | 0.52 | 0.55 | 0.48 | 0.21 | 0.18 | 0.11 | 6.66 | 7.79 | 3.62 | 0.67 | 1.16 | 0.92 |
| LOC4345159 | succinate dehydrogenase assembly factor 1 | 0.56 | 0.71 | 0.51 | 0.29 | 0.41 | 0.18 | 0.65 | 1.28 | 1.1 | 0.2 | 0.19 | 0.17 |
| LOC4346890 | succinate dehydrogenase [ubiquinone] iron-sulfur subunit 2 | 0.24 | 0.25 | 0.34 | 0.17 | 0.09 | 0.07 | 0.64 | 0.36 | 0.47 | 3.07 | 1.75 | 2.19 |
| LOC4343404 | succinate dehydrogenase subunit 3-2 | 2.41 | 1.86 | 2.32 | 2.97 | 3.85 | 4.44 | 0.44 | 0.24 | 0.5 | 4.9 | 5.76 | 5.32 |
| LOC4342350 | succinate dehydrogenase [ubiquinone] flavoprotein subunit | 5.68 | 4.97 | 3.36 | 6.45 | 4.73 | 8.96 | 2.5 | 2.01 | 3.07 | 1 | 1.06 | 1.36 |
| LOC4328125 | succinate dehydrogenase subunit 3-1 | 0.84 | 0.93 | 1.02 | 0.67 | 1.18 | 1.28 | 18.26 | 13.59 | 8.94 | 0.03 | 0.02 | 0.03 |
| LOC4350629 | succinate dehydrogenase assembly factor 2 | 0.46 | 0.32 | 0.28 | 1.88 | 4.07 | 2.29 | 5.1 | 5.47 | 8.96 | 0.9 | 0.81 | 0.67 |
| LOC4344494 | succinate dehydrogenase subunit 6 | 1.68 | 1.49 | 2.4 | 0.17 | 0.34 | 0.2 | 0.5 | 0.85 | 0.78 | 0.1 | 0.07 | 0.09 |
| LOC4335816 | succinate dehydrogenase subunit 5 | 0.24 | 0.28 | 0.21 | 0.91 | 0.33 | 0.67 | 1.42 | 1.96 | 1.44 | 0.15 | 0.14 | 0.01 |
| LOC112939253 | succinate dehydrogenase subunit 8B | 0.74 | 0.87 | 1.3 | 2.99 | 4.75 | 5.39 | 3.05 | 4.28 | 6.04 | 0.42 | 0.22 | 0.32 |
| LOC112939252 | succinate dehydrogenase subunit 8A | 1.33 | 1.49 | 0.92 | 0.03 | 0.13 | 0.09 | 0.54 | 0.75 | 0.63 | 0.09 | 0.08 | 0.16 |
| LOC4346939 | succinate dehydrogenase subunit 7 | 1.55 | 1.8 | 1.56 | 0.55 | 0.35 | 0.38 | 2.11 | 1.34 | 0.64 | 0.24 | 0.14 | 0.14 |
| LOC4332774 | fumarate hydratase 1 | 1.53 | 1.41 | 1.63 | 0.77 | 0.49 | 0.29 | 0.61 | 0.56 | 0.65 | 0.2 | 0.11 | 0.17 |
| LOC4326249 | malate dehydrogenase | 0.16 | 0.2 | 0.3 | 5.32 | 7.78 | 8.76 | 1.91 | 0.88 | 1.64 | 0.04 | 0.06 | 0.03 |
| LOC4336595 | malate dehydrogenase | 1.09 | 1.39 | 0.89 | 0.67 | 0.97 | 0.55 | 4.51 | 3.22 | 4.84 | 1.34 | 0.99 | 1.3 |
| LOC4339682 | malate dehydrogenase | 0.54 | 0.44 | 0.31 | 2.92 | 3.6 | 5.42 | 3.55 | 4.43 | 3.72 | 0.46 | 0.34 | 0.27 |
