# Supplementary figures and images for: Metabolomics Analysis in Different Development Stages on SP0 Generation of Rice Seeds After Spaceflight
Source: Front Plant Sci. 2021 Jun 30;12:700267. doi: 10.3389/fpls.2021.700267 (PMC8278407; doi:10.3389/fpls.2021.700267)

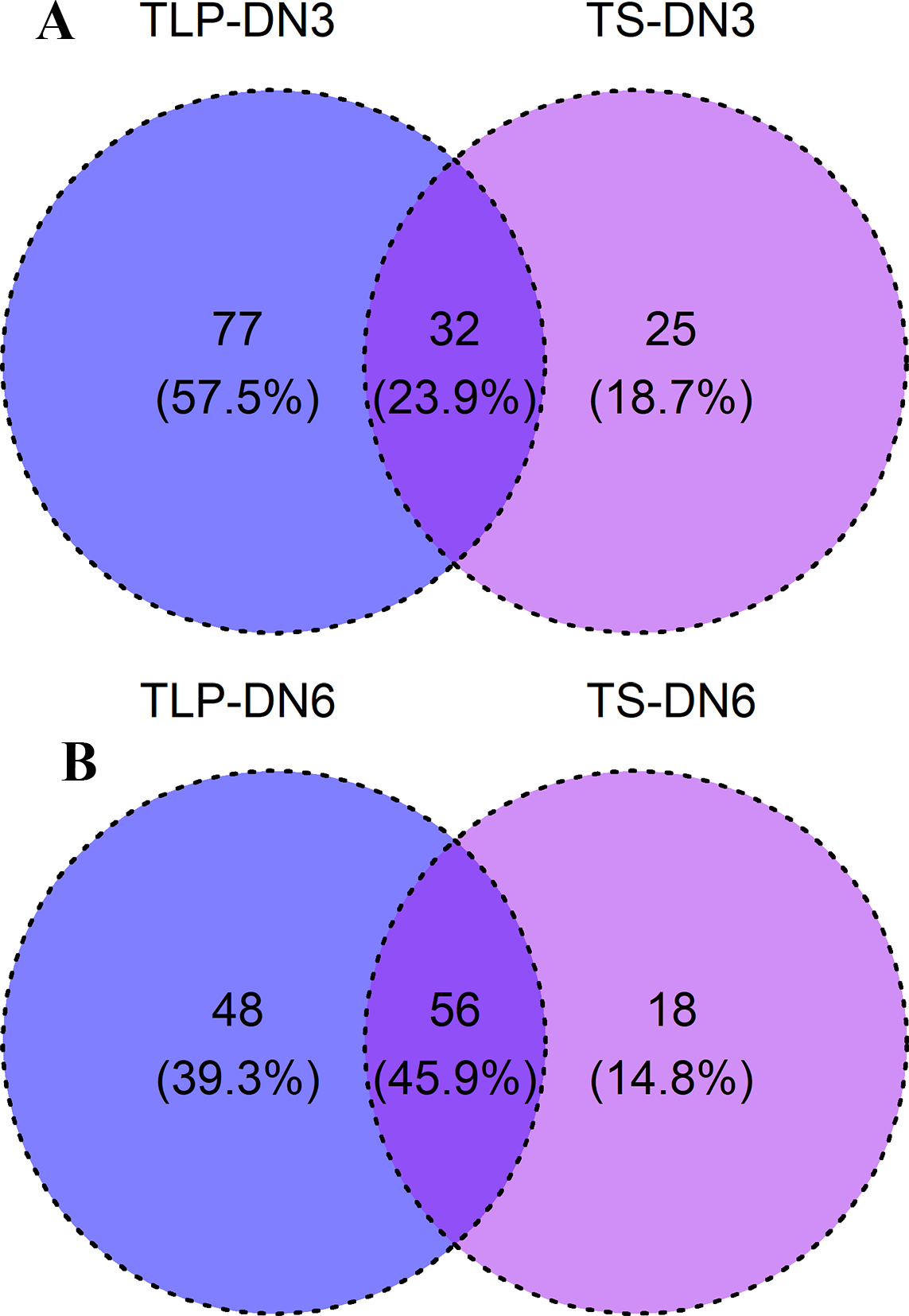

Supplement: Supplementary file 7 [file Image_1.tif]
